# Lysyl oxidase-like 3 is required for melanoma cell survival by maintaining genomic stability

**DOI:** 10.1038/s41418-017-0030-2

**Published:** 2017-12-11

**Authors:** Patricia G. Santamaría, Alfredo Floristán, Bárbara Fontanals-Cirera, Alberto Vázquez-Naharro, Vanesa Santos, Saleta Morales, Lourdes Yuste, Héctor Peinado, Antonio García-Gómez, Francisco Portillo, Eva Hernando, Amparo Cano

**Affiliations:** 1 Departamento de Bioquímica, UAM, Instituto de Investigaciones Biomédicas “Alberto Sols” CSIC-UAM, Arzobispo Morcillo 4, 28029 Madrid, Spain; 2 Centro de Investigación Biomédica en Red de Cáncer (CIBERONC), Madrid, Spain; 3 Department of Pathology and Interdisciplinary Melanoma Cooperative Group, New York University Langone Medical Center, New York, NY USA; 4 Microenvironment and Metastasis Laboratory, Department of Molecular Oncology, Spanish National Cancer Research Center (CNIO), Madrid, Spain; 5 Chromatin and Disease Group, Cancer Epigenetics and Biology Programme (PEBC), Bellvitge Biomedical Research Institute (IDIBELL), Barcelona, Spain

## Abstract

Lysyl oxidase-like 3 (LOXL3) is a member of the lysyl oxidase family comprising multifunctional enzymes with depicted roles in extracellular matrix maturation, tumorigenesis, and metastasis. In silico expression analyses followed by experimental validation in a comprehensive cohort of human cell lines revealed a significant upregulation of LOXL3 in human melanoma. We show that LOXL3 silencing impairs cell proliferation and triggers apoptosis in various melanoma cell lines. Further supporting a pro-oncogenic role in melanoma, LOXL3 favors tumor growth *in vivo* and cooperates with oncogenic BRAF in melanocyte transformation. Upon LOXL3 depletion, melanoma cells display a faulty DNA damage response (DDR), characterized by ATM checkpoint activation and inefficient ATR activation leading to the accumulation of double-strand breaks (DSBs) and aberrant mitosis. Consistent with these findings, LOXL3 binds to proteins involved in the maintenance of genome integrity, in particular BRCA2 and MSH2, whose levels dramatically decrease upon LOXL3 depletion. Moreover, LOXL3 is required for efficient DSB repair in melanoma cells. Our results reveal an unexpected role for LOXL3 in the control of genome stability and melanoma progression, exposing its potential as a novel therapeutic target in malignant melanoma, a very aggressive condition yet in need for more effective treatment options.

## Introduction

Lysyl oxidase-like 3 (LOXL3) is a member of the lysyl oxidase (LOX) protein family that comprises five closely related members, prototypical LOX and four LOX-like enzymes (LOXL1–4) [1]. LOX proteins are primarily known for their role as extracellular enzymes; upon secretion they promote stabilization of collagen and elastin fibers contributing to extracellular matrix (ECM) maturation [2–4]. Beyond ECM cross-linking, lysyl oxidases have been involved in gene transcription, epithelial to mesenchymal transition (EMT), development, differentiation, and angiogenesis, as well as in distinct pathologies such as fibrosis and cancer (reviewed in refs. [5–8]). Some of the emerging roles of several LOX members are independent of their secretion and have been associated with their intracellular and intranuclear localization [5, 6, 8, 9]. Moreover, the amine oxidase catalytic activity is not always required for some of the recently reported lysyl oxidase functions, including their involvement in angiogenesis, EMT, and inflammation [6, 9–11], suggesting complex and wide-ranging roles for the members of the LOX family.

Human LOXL3 presents differential tissue expression regarding other LOX proteins [12–15] and has been recently proposed as a candidate gene responsible for recessive autosomal Stickler syndrome [16], a collagenopathy [17], whereas null mutations in *LOXL3* have been associated with early-onset high myopia [18]. Genetic inactivation in mice has revealed Loxl3 involvement in skeletal, muscular, and lung development [19–21]. Thus far the reported roles for LOXL3 were essentially associated with LOXL3 extracellular activity regarding ECM maturation, whereas LOXL3 involvement in cancer remains limited. Our previous studies identified human LOXL3 as modulator of EMT and Snail1 functional activity [22] and LOXL3 has been involved in the nuclear regulation of Stat3 activity [9]. Since the involvement of several LOX members in cancer has been extensively characterized [5, 6, 8], we explored LOXL3 contribution to human cancer by interrogating a comprehensive set of human cancer samples for LOXL3 expression that unveiled an association of LOXL3 to melanoma. We, therefore, performed gain and loss-of-function experiments to determine the contribution of LOXL3 to melanoma pathogenesis. Our studies reveal that human melanoma cells are addicted to LOXL3 expression since LOXL3 knockdown halts cell proliferation and triggers apoptosis. Moreover, LOXL3 cooperates to malignant transformation and contributes to melanomagenesis. We have found that LOXL3 binds to proteins that protect genome integrity (BRCA2, MSH2, SMC1A, NUMA1) and that its absence promotes a defective DNA damage checkpoint activation, deficient DNA repair and aberrant mitosis in melanoma cells. Our data uncover an unprecedented role for LOXL3 in melanoma biology and support the relevance of LOXL3 as a novel druggable target for therapeutic intervention in this severe disease.

## Results

### LOXL3 is overexpressed in human melanoma

**Fig. 1:**
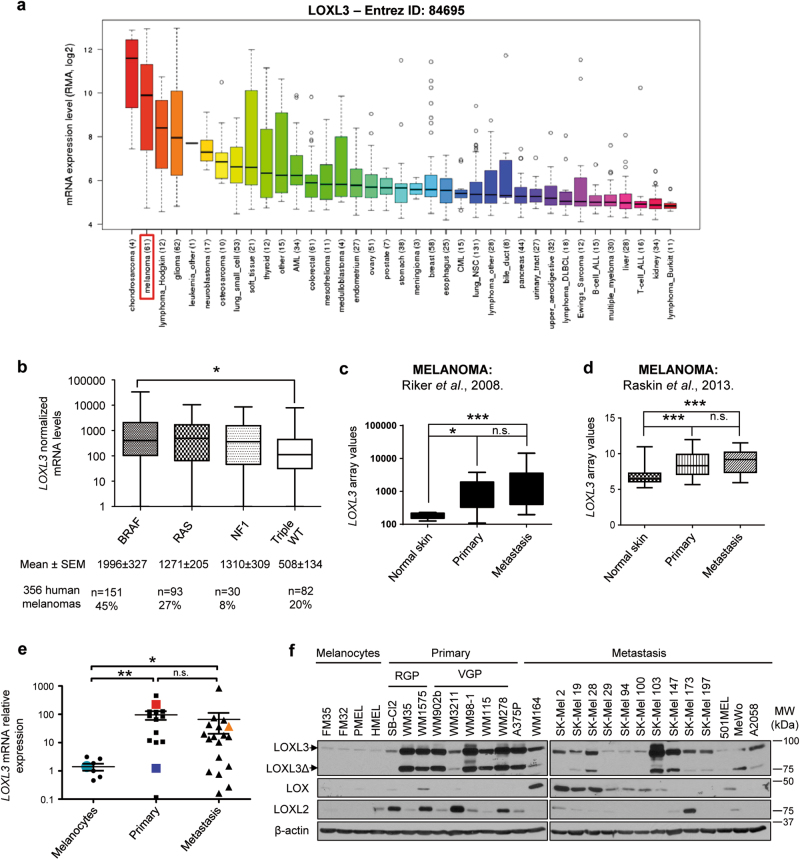
LOXL3 expression levels in human melanoma derived samples. **(a)** *LOXL3* gene expression levels in human tumor cell lines of the Broad Institute’s Cancer Cell Line Encyclopedia (CCLE). The number of cell lines analyzed for each tumor type is annotated. (**b)** *LOXL3* normalized mRNA levels within the different genomic subtypes for melanoma classification determined by The Cancer Genome Atlas (TCGA) [24] and defined by significantly mutated genes in a large sample cohort (*n* = 356 patients). Error bars represent s.e.m. ∗*p* < 0.05 by one-way ANOVA (**c**) *LOXL3* mRNA relative levels from microarray analysis comparing 40 metastatic melanoma samples, 16 primary melanoma samples, 4 samples of normal human skin, and 1 sample of human epithelial melanocytes (NHEM). ∗*p* < 0.05, ∗∗∗*p* < 0.001 by a Mann–Whitney test. (**d)** *LOXL3* mRNA relative levels from microarray analysis comparing 76 samples (46 primary melanomas, 14 melanoma metastasis, and 16 normal skin samples). ∗∗∗*p* < 0.001 by a two-sided Student’s *t*-test. (**e**) *LOXL3* mRNA expression levels relative to *GAPDH* mRNA analyzed by qPCR (8 melanocyte control cell lines, 14 primary melanomas, and 18 metastatic melanomas, indicated in Supplementary Table 1). *LOXL3* mRNA expression in HMEL (light blue), 501MEL (dark blue), MeWo (light orange), and A375P (red) cells is indicated. ∗*p* < 0.05, ∗∗*p* < 0.01 by a Mann–Whitney test. n.s.: not statistically significant (**c**–**e**). **(f**) LOXL3, LOXL2, and LOX protein levels analyzed by western blot in primary melanocytes (FM35 and FM32), immortalized melanocytes (HMEL and PMEL), and human melanoma cell lines derived from primary or metastatic sites as indicated. LOXL3 and LOXL3Δ isoforms with different mobility upon western blot analysis are shown. β-actin was used as a loading control. RGP radial growth phase, VPG vertical growth phase. Molecular weight (MW) of protein standards (kDa) is indicated

In order to explore the involvement of LOXL3 in cancer, we performed *in silico* analyses of public data sets. Mining of the Cancer Cell Line Encyclopedia (CCLE) database (www.broadinstitute.org/ccle) rendered *LOXL3* gene expression levels in 1036 cancer cell lines [23]. Besides Hodgkin lymphoma and glioma, LOXL3 highest expression was found in melanoma and chondrosarcoma (Fig. 1a). Considering *LOXL3* mRNA expression levels and the cell lines corresponding to each tumor type analyzed (*n* = 61 for melanoma, *n* = 4 for chondrosarcoma) [23] we further evaluated LOXL3 role in melanoma. Data mining of The Cancer Genome Atlas (TCGA), including whole genome sequencing and transcriptomic profiles of more than 300 cutaneous melanoma patients [24], revealed upregulated *LOXL3* mRNA levels significantly associated with the presence of well-known driver mutations in melanoma. In particular, *LOXL3* levels were higher among tumors harboring *BRAF* activating mutations whereas a similar trend, without reaching statistical significance, was observed for the *NRAS* and the *NF1* subtype compared to the *LOXL3* mRNA levels found in the triple WT subtype (Fig. 1b). The latter comprised all those samples not harboring mutations in any of the aforementioned genes, suggesting a link between canonical MAPK pathway activation and *LOXL3* upregulation in melanoma. Moreover, examination of published melanoma transcriptomic profiles [25, 26] and TCGA data [24] for *LOXL3* expression confirmed a significant upregulation of *LOXL3* levels in both primary and metastatic melanoma patient-derived samples compared to controls (Fig. 1c, d), while no differences in expression were found between primary and metastatic samples (Fig. 1c, d; Supplementary Fig. 1a). Accordingly, analysis of a large cohort of primary and metastatic melanoma cell lines (Supplementary Table 1) confirmed increased LOXL3 expression at both LOXL3 mRNA and protein levels in melanoma cells compared to primary and immortalized human melanocytes (Fig. 1e, f). As depicted in Fig. 1f, canonical LOXL3 and a splicing variant lacking exons 4 and 5 (LOXL3ΔE4E5 or LOXL3Δ) (Supplementary Fig 1b, c), were detected by western blot. Both LOXL3 isoforms were found similarly expressed in most melanoma cells tested (Fig. 1f). LOXL3Δ isoform probably corresponds to the recently identified splice variant LOXL3-sv2 [27]. LOXL3 expression is thus associated with both primary and metastatic melanomas, and considerably upregulated compared to other LOX family members frequently associated with cancer, such as LOX and LOXL2 [5, 6] (Fig. 1f). These results suggest a previously unexplored role for LOXL3 in melanoma.

### Silencing LOXL3 expression in melanoma induces cell death

**Fig. 2:**
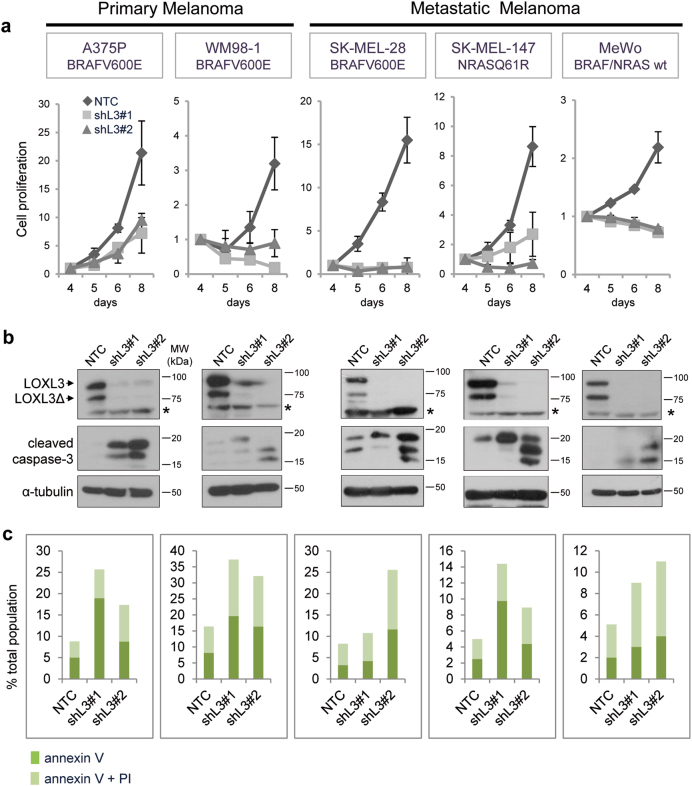
Melanoma cell lines are addicted to LOXL3 expression. **(a**) Cell proliferation of human melanoma cell lines transduced with lentiviral particles expressing control (NTC) and two different LOXL3 targeting shRNAs (shL3#1 and shL3#2). Cells were infected and then selected with 1 μg/ml of puromycin for 2 days before cell proliferation was analyzed; time points in the *x*-axis indicate days post-infection (dpi). Representative cell proliferation curves are shown for each cell line with three experimental replicates for each time point; error bars represent s.e.m. The experiments were repeated at least three times with similar results. (**b**) Downregulation of LOXL3 protein levels was verified by western blot, as well as the cleavage of caspase-3 (middle panels), indicative of apoptosis induction, in all the cell lines analyzed. Cells were infected and selected as in **a** and attached and floating cells were collected 4–6 dpi for total protein extraction. α-tubulin was used as a loading control. LOXL3 and LOXL3Δ isoforms are indicated in the upper panels. Asterisks (*) indicate a non-specific band recognized by LOXL3 antibody. MW of protein standards (kDa) is indicated. **(c**) Percentage of apoptotic cells in the different melanoma cell lines upon lentiviral infection with the indicated shRNAs as determined by FACS analysis of annexin V binding and propidium iodide (PI) uptake. Cells were infected as in (**a**) and collected as in (**b)** before annexin V/PI incubation. The percentage of annexin V positive and double-positive cells for both annexin V and PI is indicated. One representative experiment out of three is shown

To investigate LOXL3 involvement in melanoma, we initially evaluated the effects of LOXL3 silencing in a panel of human melanoma cells. We designed two different short hairpin RNAs (shRNAs) against LOXL3 also targeting LOXL3Δ without altering LOX and LOXL2 protein levels (Supplementary Fig. 2a). All melanoma cell lines depleted for LOXL3 expression halt proliferation between 5–8 days post-infection (dpi) compared to control cells infected with a non-targeting control (NTC) (Fig. 2a). LOXL3 depletion had analogous effects on cells harboring *BRAF* (A375P, WM98-1, and SK-MEL-28) or *NRAS* (SK-MEL-147) activating mutations as well as on cells wild type for *NRAS/BRAF* lacking NF1 protein (MeWo) [28] (Fig. 2a and Supplementary Table 1). Moreover, LOXL3 silencing was deleterious for primary (A375P and WM98-1) and metastatic melanoma cell lines (SK-MEL-28, SK-MEL-147, and MeWo) corroborating that melanoma cells require LOXL3 expression to survive independently of their mutational status or the disease stage from which they were derived. We then analyzed whether the absence of LOXL3 prevents cell growth by triggering apoptosis. Indeed, LOXL3-depleted cell lines displayed caspase-3 activation (Fig. 2b) and increased annexin V staining (Fig. 2c) at 5–6 dpi compared to control NTC-transduced cells. Likewise, efficient LOXL3 silencing by transient siRNA transfection induced a similar level of apoptosis as shRNA transduction in SK-MEL-28 cells as shown by caspase-3 cleavage and annexin V staining (Supplementary Fig. 2b). Moreover, the CRISPR/Cas9-mediated knockout of LOXL3 in A375P cells also promoted a cell proliferation delay accompanied by an increase in apoptotic cell death (Supplementary Fig. 2c–e). Taken together, we conclude that human melanoma cell lines expressing LOXL3 are addicted to the maintenance of its expression.

### LOXL3 promotes tumorigenesis

**Fig. 3:**
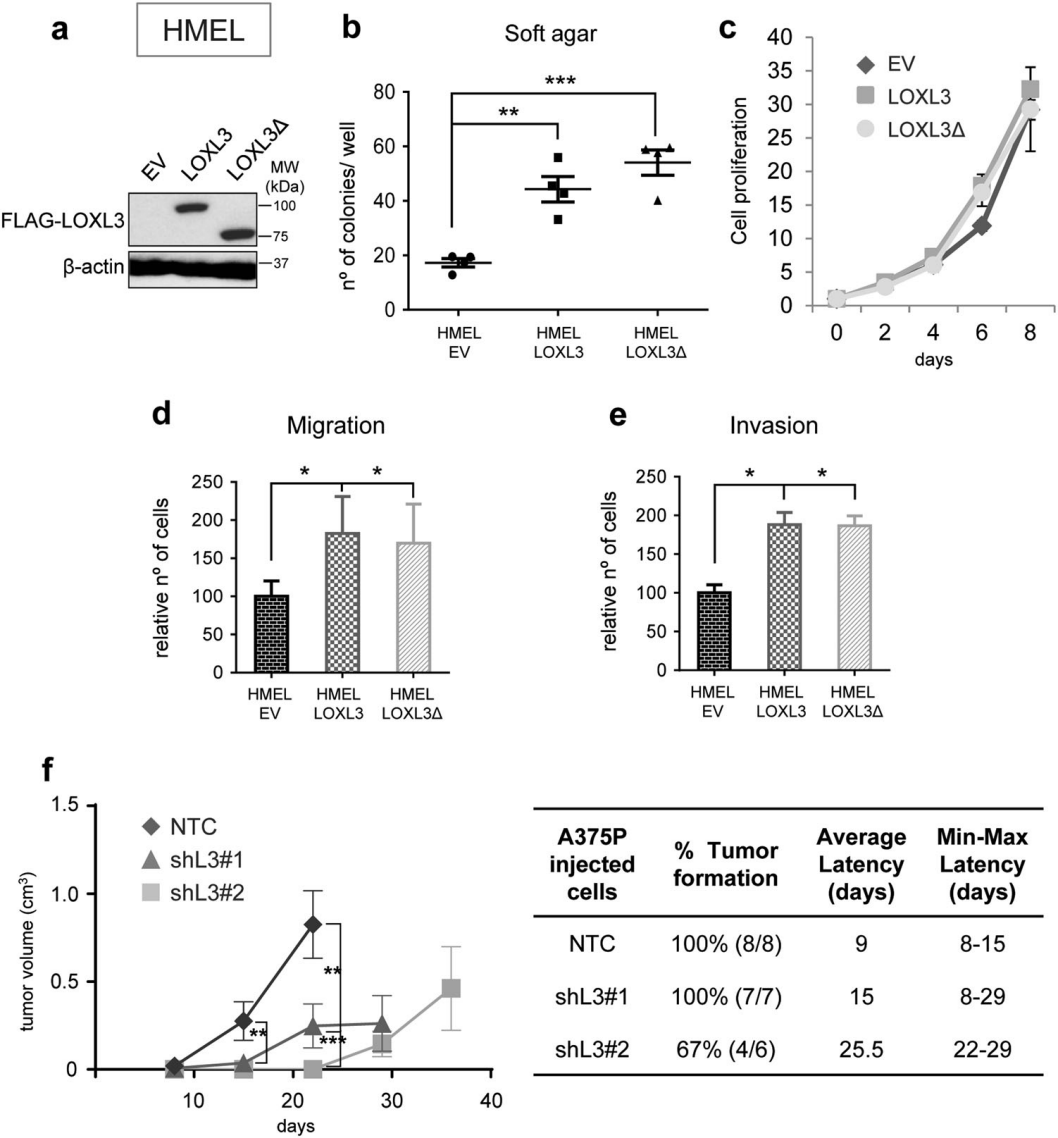
LOXL3 contributes to transformation of immortalized melanocytes and favors tumor growth. ** (a)** HMEL immortalized melanocytes were infected with GFP lentiviral particles expressing empty vector (EV), FLAG-LOXL3, or FLAG-LOXL3Δ and GFP-positive sorted cells were analyzed for the expression of LOXL3 isoforms by western blot. β-actin was used as a loading control. MW of protein standards (kDa) is indicated. (**b**) Soft-agar colony formation assay of HMEL melanocytes transduced as in **(a)**. Error bars represent s.e.m., *n* = 4 biologically independent replicates. (**c)** Cell proliferation of HMEL melanocytes transduced as in **a**. Cell proliferation is shown for each cell line with three experimental replicates; error bars represent s.e.m. The experiment was repeated twice with similar results. **(d** and **e)** HMEL immortalized melanocytes transduced as in (**a)** were analyzed in migration **(d)** and invasion **(e)** assays. Graphs show the number of migrating and invading cells quantified after cell fixation and staining. Error bars represent s.d., *n* = 3 biologically independent experiments. **(f**) A375P NTC, shL3#1 and shL3#2 xenografts were grown in nude mice (*n* = 8 for NTC, *n* = 7 for shL3#1 and *n* = 6 for shL3#2) and tumor volume was measured every week (left). Error bars represent s.e.m. The percentage of tumors developed (incidence) in each group and the required days from cell injection until the tumors were measurable (latency) is shown on the middle. The average latency and minimal and maximal days for latency in each group is also indicated (right). ∗*p* < 0.05, ∗∗*p* < 0.01, ∗∗∗*p* < 0.001 by a two-sided Student’s *t*-test (**b** and **d**–**f**)

To assess LOXL3 contribution to melanomagenesis, we overexpressed LOXL3 in immortalized melanocytes expressing BRAFV600E (HMEL) [29] with insignificant levels of endogenous LOXL3 (Fig. 1f and Fig. 3a). This cell line allows investigating the cooperation of genetic alterations to neoplastic transformation. Stable LOXL3 and LOXL3Δ expression in HMEL cells greatly enhanced their anchorage independent growth, a hallmark of transformation (Fig. 3b) without affecting cell proliferation (Fig. 3c). Additionally, LOXL3 and LOXL3Δ ectopic expression favored migration and invasiveness of HMEL cells (Fig. 3d, e). Similarly, LOXL3 transduction in a metastatic melanoma cell line (501MEL) harboring minimal endogenous LOXL3 levels (Fig. 1f and Supplementary Fig. 3a) promoted migration and invasion without affecting cell proliferation (Supplementary Fig. 3b–d), whereas endogenous LOXL3 silencing had analogous effects as previously shown for other melanoma cell lines (Supplementary Fig. 3e–g). Besides, tumorigenic A375P cells depleted for LOXL3 (Fig. 2b) showed increased latency and delayed tumor growth compared to control cells (Fig. 3f). Thus, enhanced LOXL3 expression cooperates with oncogenic alterations such as active BRAF to transform human melanocytes favoring migration and invasion of melanoma cells and supporting melanoma tumorigenicity.

### LOXL3 binding partners are involved in mitosis and the DNA damage response (DDR)

**Fig. 4:**
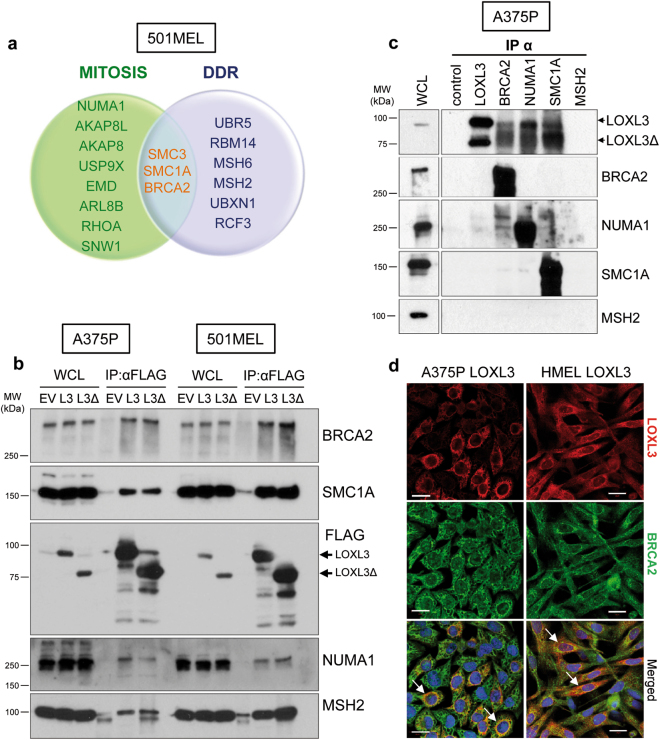
LOXL3 physically interacts with proteins involved in genomic integrity maintenance. **(a)** Venn diagram showing common proteins identified by mass spectrometry related to mitosis (green) and DDR (lilac) in 501MEL cells overexpressing LOXL3 or LOXL3Δ. Proteins involved in both processes are indicated in the middle (orange letters). **(b)** Interaction of selected proteins was confirmed by immunoblot analyses in A375P and 501MEL cells stably expressing empty vector (EV), FLAG-tagged LOXL3 (L3) or LOXL3Δ isoform (L3Δ) after FLAG immunoprecipitation. Whole-cell lysates (WCL) and eluted samples upon FLAG immunoprecipitation (IP: α-FLAG) were loaded and western blot analyses with the indicated antibodies were performed. Data are representative of three independent experiments. **(c)** Analysis of endogenous protein binding was performed in A375P whole-cell lysates (WCL) by individual immunoprecipitation (IP) with specific antibodies against BRCA2, NUMA1, SMC1A, and MSH2. WCL, control (IP without antibody) and eluates from the indicated samples were analyzed by western blot for the presence of LOXL3. MSH2 was not immunoprecipitated. MW of protein standards (kDa) is indicated (**b**, **c**). **(d**) Confocal images showing co-localization of 55 and 33% (Manders’ Co-localization Coefficient) of LOXL3 and BRCA2 in A375P and HMEL cells, respectively. Cell lines were stably transduced with FLAG-LOXL3, fixed and processed for BRCA2 (green) and FLAG (red) immunofluorescence. DAPI (blue) was used to stain DNA. White arrows in the merged images mark the subcellular location where BRCA2 and LOXL3 co-localize. Scale bar: 20 μm

We carried out a comprehensive proteomic analysis of LOXL3 protein interactors to further understand LOXL3 involvement in melanomagenesis. Since we could not discriminate specific roles for LOXL3 and LOXL3Δ, we ectopically expressed both FLAG-tagged isoforms in metastatic melanoma 501MEL cell line (Supplementary Fig. 3a) and performed an anti-FLAG immunopurification followed by mass spectrometry analysis. After analysis and filtering, we focused on those common proteins identified in eluates from LOXL3 and LOXL3Δ transduced cells, falling a number of them (with 5 to 64 identified peptides) within the functional categories of mitosis and/or DDR (Fig. 4a and Supplementary Table 2). To validate the interactions, we performed immunoprecipitation experiments in 501MEL and A375P melanoma cells expressing ectopic LOXL3 or LOXL3Δ FLAG-tagged proteins. We detected specific binding of LOXL3 to BRCA2, SMC1A, MSH2, and NUMA1 (Fig. 4b), proteins involved in DDR and/or mitosis [30–33], suggesting a role for LOXL3 in these biological processes. Binding of endogenous LOXL3 to endogenous BRCA2, NUMA1, and SMC1A proteins was also confirmed (Fig. 4c and Supplementary Fig. 4a). LOXL3 localization has been described as cytoplasmic/perinuclear [22] and nuclear [9]. Consistent with LOXL3 binding to recognized nuclear partners, cellular fractionation experiments in A375P cells revealed LOXL3 presence in the nuclear compartment (Supplementary Fig. 4b). Further differential centrifugation experiments located LOXL3 and its binding partners in the same enriched cytoplasmic and nuclear subcellular fractions (Supplementary Fig. 4c) supporting their biological association. Moreover, confocal microscopy analyses determined LOXL3 and BRCA2 co-localization in the cytoplasm and perinuclear area of melanoma cells (Fig. 4d).

### Melanoma cells activate the DDR upon LOXL3 knockdown

**Fig. 5:**
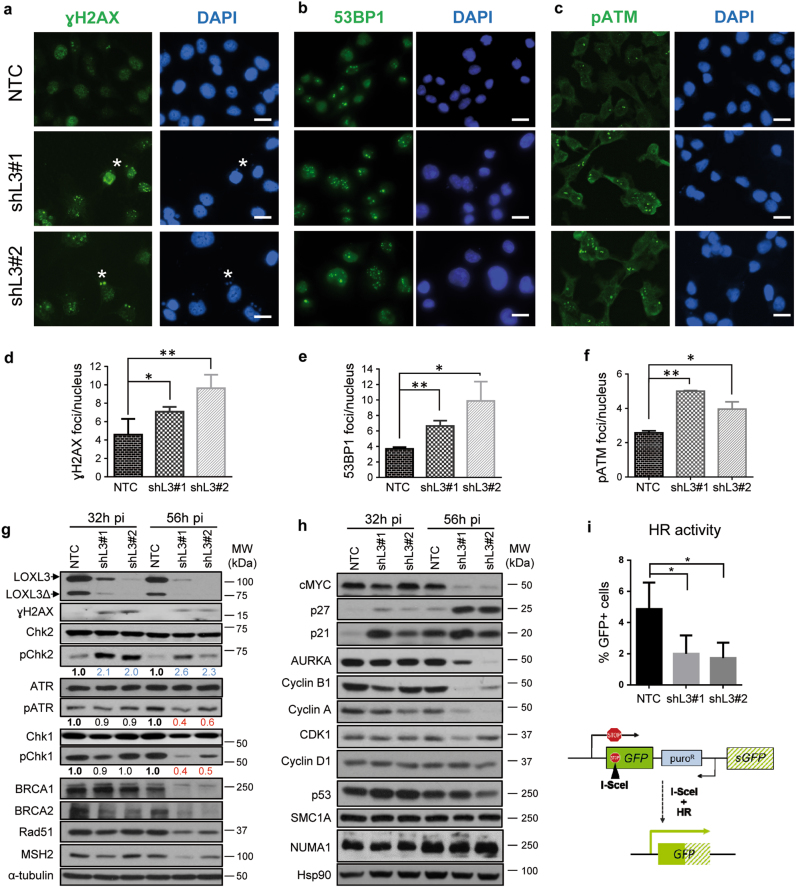
LOXL3 is involved in the DNA damage checkpoint. **(a–c**) A375P cells were transduced with control (NTC) and LOXL3 targeting (shL3#1 and shL3#2) lentivirus and collected at 56 h pi, fixed and processed for γH2AX (**a**), 53BP1 (**b**), and phospho-ATM (pATM) (**c**) immunofluorescence. DAPI (blue) was used to stain DNA. Representative micrographs are shown. White asterisks **a** mark the presence of γH2AX micronuclei. Scale bar: 20 μm. (**d-f**) Quantitation of γH2AX (**d**), 53BP1 (**e**) and pATM (**f**) immunofluorescence foci. Error bars represent s.e.m., *n* = 5 biologically independent replicates for γH2AX (1000 total cells analyzed), *n* = three biological replicates for 53BP1 (450 total cells analyzed) and *n* = 2 biological replicates for pATM (500 total cells analyzed). ∗*p* < 0.05, ∗∗*p* < 0.01 by a two-sided Student’s *t*-test. **(g** and **h**) Whole-protein extracts from control (NTC) and LOXL3-silenced (shL3#1 and shL3#2) A375P cells after 32 and 56 h pi were subjected to western blot analyses with the indicated antibodies. AURKA: Aurora Kinase A. α-tubulin (**g**) and Hsp90 (**h**) were used as loading controls. The phospho-ATR (pATR), phospho-Chk1 (pChk1), and phospho-Chk2 (pChk2) levels were calculated normalizing to the levels of the corresponding total proteins using ImageJ and their relative levels regarding NTC control cells are indicated below the matching immunoblots. Data shown are representative of three biologically independent replicates. MW of protein standards (kDa) is indicated (**g,** **h**). (**i)** HR activity represented as the percentage of GFP-positive cells analyzed by FACS from A375P pHPRT-DRGFP cells transduced with control (NTC) and LOXL3 shRNA lentivirus (shL3#1 and shL3#2) after I-SceI transfection. Error bars represent s.d., *n* = 4 biologically independent experiments. ∗*p* < 0.05 by a two-sided Student’s *t*-test. Lower panel displays a scheme summarizing the experiment performed. pHPRT-DRGFP reporter consists of two defective *GFP* genes, the first contains an I-SceI endonuclease site. Subsequent cellular expression of I-SceI leads to a DSB that can be repaired by HR using the downstream wild-type GFP sequence as a template, resulting in GFP-positive cells

The interaction of LOXL3 with proteins involved in repairing damaged DNA at double-strand breaks (DSBs) (Fig. 4) led us to hypothesize the association of LOXL3 to DDR checkpoint activation and/or DSB repair. LOXL3-silenced A375P cells accumulated phosphorylated H2AX (γH2AX) foci (Fig. 5a, d, g), a marker of DSBs [34], as well as 53BP1 foci (Fig. 5b, e), a mediator of DSB repair (reviewed in ref. [35]) to similar levels to those induced by known DNA damage agents, such as hydroxyurea or ionizing radiation (Supplementary Fig. 5a, b). Noticeably, at early time points post-lentiviral infection (32–56 h pi), and before any effects on cell proliferation nor apoptosis were detected (Fig. 2 and Supplementary Fig. 6a), LOXL3-silenced cells showed activation of ATM (Fig. 5c, f and Supplementary Fig. 5c), a master checkpoint kinase activated after DSBs (Supplementary Fig. 5d) and required for DNA repair and genome stability [36]. Concomitantly, LOXL3 silencing promoted phosphorylation of the ATM effector checkpoint substrate, Chk2 (Fig. 5g). In contrast, activation of ATR, another central kinase mediating DDR [36], is reduced shortly after LOXL3 knockdown, as levels of pATR and its immediate target, Chk1, remarkably fell upon LOXL3 silencing (Fig. 5g). In fact, the levels of other relevant downstream effectors of ATM and ATR pathways that ultimately mediate the repair of DNA damage such as BRCA1, BRCA2, Rad51, and MSH2 [37, 38], dropped shortly after LOXL3 depletion (Fig. 5g and Supplementary Fig. 5e), whereas NUMA1 and SMC1A levels remained stable (Fig. 5h). p53 and p21 levels increased initially consistent with DDR activation [36] but p53 levels were not steadily maintained afterwards (Fig. 5h). Indeed, cells depleted for LOXL3 displayed reduced ability to repair DSBs as shown using the DRGFP reporter to measure the frequency of homologous recombination (HR) [39] (Fig. 5i) while non-homologous end joining (NHEJ) [40] repair pathway was not altered (Supplementary Fig. 5f). Altogether, these results suggest that LOXL3 expression in melanoma cells allows for DNA damage repair by maintaining the protein levels of BRCA2, Rad51, and MSH2. Besides, upon LOXL3 silencing, melanoma cells accumulate DSBs and subsequently activate the ATM checkpoint kinase but fail to induce a complete DDR.

### LOXL3 is required for proper mitotic completion

**Fig. 6:**
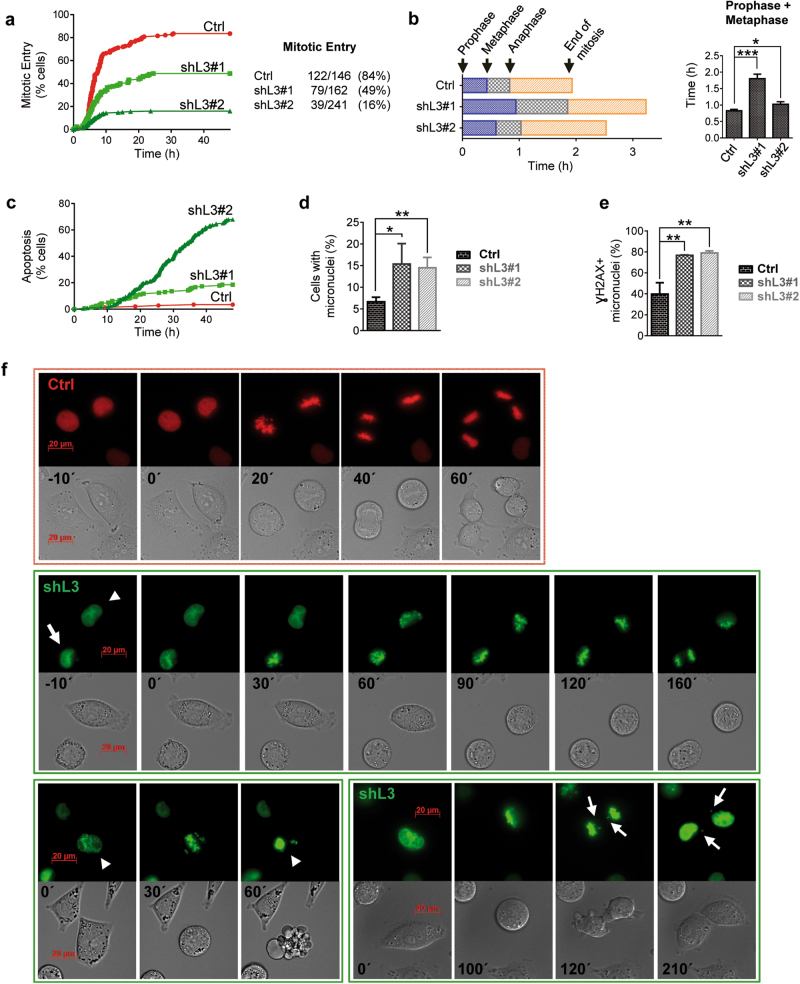
LOXL3 is required for proper mitotic completion. **(a)** A375P cells transduced with scramble (Ctrl, H2B-RFP, red) and LOXL3 targeting (shL3#1 and shL3#2, H2B-GFP, green) lentivirus were synchronized in G1/S with 2.5 mM thymidine for 20 h and upon release their mitotic progression was followed by high-resolution fluorescence video microscopy and automated image analysis. The graph (left) shows the percentage of cells that enter mitosis starting 4 h after thymidine release and analyzed for 48 h. The total number of cells that were analyzed in each cell culture and the total number and percentage of those cells that enter mitosis is indicated (right). The experiment was repeated twice with similar results. **(b**) Graph representing the length of each mitotic phase measured in the cells recorded in (**a)** from control (Crtl) and LOXL3-silenced cells (shL3#1 and shL3#2) (left). The combined lengths of prophase and metaphase is significantly longer in cells transduced with shL3#1 and shL3#2 compared to A375P control cells (right). **(c)** Graph depicting the percentage of control (Ctrl) and LOXL3-depleted cells (19% of shL3#1 and 68% of shL3#2) that suffer apoptosis during the 48 h time-lapse imaging analyzed after thymidine block release. **(d)** Graph showing the percentage of control and LOXL3-depleted cells containing micronuclei (*n* > 100 for control and *n* > 300 for LOXL3-silenced cells). **(e)** Control (Ctrl) and LOXL3-silenced A375P cells (shL3#1 and shL3#2) were collected at 56 h pi, fixed and processed for γH2AX immunofluorescence. Graph shows the percentage of γH2AX positive micronuclei present in these cells (*n* > 50 for control and *n* > 250 for LOXL3-silenced cells). Error bars represent s.e.m. (**f)** Representative images from fluorescence time-lapse imaging from control (red) and LOXL3-depleted A375P cells (green) depicting examples of regular mitosis (upper panel), lengthier mitosis (middle panel, cell marked with an arrow), mitotic abnormalities (middle panel, cell marked by an arrowhead), mitotic catastrophes (lower panel, left, cell marked by an arrowhead) and lagging chromosomes, resulting in micronuclei formation (lower panel, right, cell marked by arrows). Minutes indicating the elapsed time from the start of chromosome condensation are shown inside phase-contrast images for each individual panel. Scale bar: 20 μm. ∗*p* < 0.05, ∗∗*p* < 0.01, ∗∗∗*p* < 0.001 by a two-sided Student’s *t*-test **(b**, **d**, **e)**

We then analyzed the cell-cycle progression of melanoma cells and unveiled a consistent accumulation of cells in G2/M phase in the absence of LOXL3 compared to control cells (Supplementary Fig. 6a). At the molecular level, upon LOXL3 silencing, MYC levels decreased along with p21 and p27 upregulation (Fig. 5h), and the protein levels of mitotic regulators such as AURKA, cyclin A and cyclin B1 were diminished while CDK1 and cyclin D levels did not significantly change compared to control cells (Fig. 5h). Since LOXL3-depleted melanoma cells ultimately die after shRNA transduction (Fig. 2), we analyzed how A375P cells were able to enter and exit mitosis in the absence of LOXL3 at early time points after infection. G1/S synchronized A375P control and LOXL3-depleted cells were released into fresh media to follow their cell-cycle progression by FACS. The total percentage of A375P cells that entered G2/M phase was not affected by LOXL3 silencing, whereas a significant delay in the ability of LOXL3-depleted cells to exit G2/M compared to control NTC cells was detected (Supplementary Fig. 6b, c), suggesting an essential role for LOXL3 in G2/M completion. Correspondingly, we observed that endogenous LOXL3 protein levels accumulated in cells during G2/M phase (Supplementary Fig. 6d). To further investigate cell-cycle progression, LOXL3 shRNAs and scrambled (Ctrl) shRNA were subcloned into lentiviral vectors expressing histone 2B-green fluorescent protein (H2B-GFP) or histone 2B-red fluorescent protein (H2B-RFP), respectively [41]. Control (red) and LOXL3 knockdown A375P cells (green) were synchronized in G1/S, released into fresh media and their mitotic progression was analyzed by fluorescence time-lapse imaging from 4 to 48 h. We observed that while 84% of control cells entered mitosis (122 out of 146, Fig. 6a) taking approximately 2 h for completion (Fig. 6b), only 49% of shL3#1 knockdown cells entered mitosis (79 out of 162, Fig. 6a) and progressed slower (Fig. 6b). Note that even fewer A375P cells transduced with shL3#2 were able to enter mitosis (16%, 39 out of 241, Fig. 6a). Particularly, LOXL3-silenced cells spent more time to progress to anaphase than control cells (Fig. 6b) and a remarkable fraction of LOXL3-silenced cells underwent apoptosis before entering mitosis compared to control cells (Fig. 6c). Moreover, close to 10% of LOXL3 knockdown cells attempting to divide died through mitotic catastrophe (Fig. 6f, lower panels, and Supplementary Fig. 6e). LOXL3-depleted A375P cells also displayed mitotic abnormalities such as an increased number of micronuclei (Fig. 6d, f) often related to chromosome segregation defects [42]. Besides, most of these micronuclei also accumulated γH2AX (Fig. 5a and Fig. 6e), a marker of genomic instability. Finally, LOXL3 silencing entailed faulty chromosome alignment in the metaphase plate and the presence of lagging chromosomes upon anaphase was also detected (Fig. 6f, lower panels). The alterations in cell-cycle progression can be observed in representative videos from A375P LOXL3-silenced cells compared to control cells (Supplementary Videos V1-V3). Altogether, these data indicate that the presence of LOXL3 is required for proper mitotic progression.

### Upregulation of LOXL3 in melanoma contributes to genomic stability

**Fig. 7:**
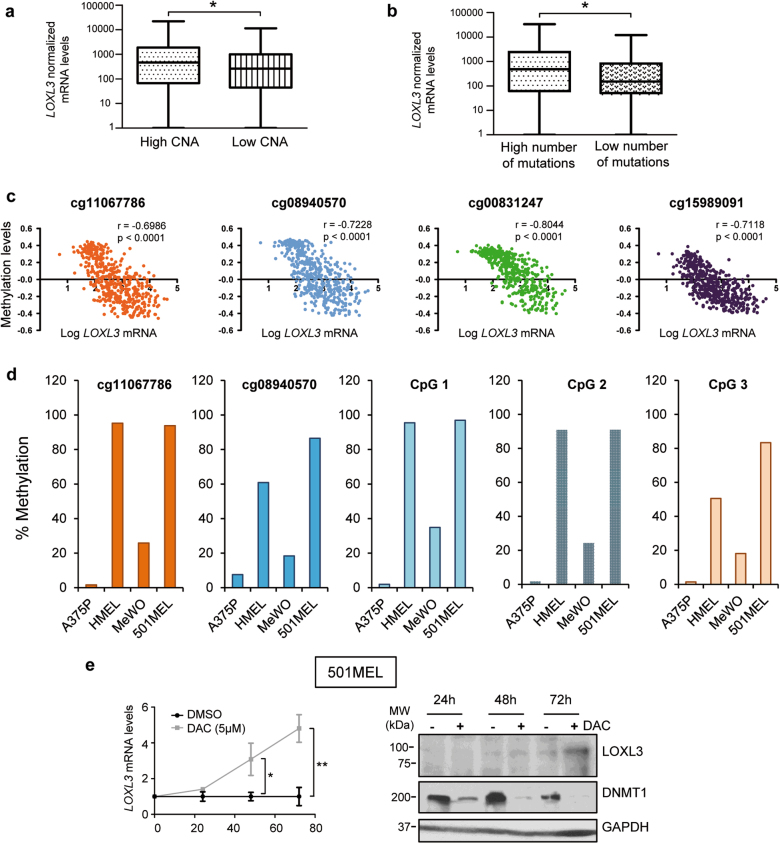
LOXL3 sustains genome stability. **(a** and,** b)** ma samples analyzed by The Cancer Genome Atlas (TCGA) [24] were classified by CNA (copy number alterations, score) **(a)** (*n* = 351 patients) and total number of mutations (**b**) (*n* = 316 patients) and plotted against *LOXL3* normalized mRNA levels (first vs. fourth quartile). Error bars represent s.e.m. ∗*p* < 0.05 by a two-tailed Student’s *t*-test. **(c**) *LOXL3* methylation levels compared to *LOXL3* mRNA expression levels in tumors from the TCGA [24] melanoma data set (*n* = 473) corresponding to CpG loci with indicated probes from TCGA [24] within regulatory regions of human *LOXL3* gene. *p*-value was calculated by two-tailed Spearman correlation. **(d**) Percentage of *LOXL3* methylation in the indicated melanoma cell lines (A375P, HMEL, MeWo, and 501MEL) within the CpG loci corresponding to two probes depicted in **c** and three additional probes designed for CpG loci located within intron 1 of human *LOXL3* gene (CpG 1–3) (see Supplementary Fig. 7). **(e)** 501MEL cells were treated with vehicle (DMSO) or 5 µM decitabine (DAC) and LOXL3 mRNA (left panel) or LOXL3 protein levels (right panel) were analyzed at the indicated time points, *n* = 3 biologically independent replicates. Left, error bars represent s.e.m. ∗∗*p* < 0.01 by a two-sided Student’s *t*-test. Right, results from one representative experiment are shown. DNMT1 was used to confirm methylation inhibition by DAC and GAPDH was used as a loading control

Due to the aforementioned results involving LOXL3 in DNA repair mechanisms and mitosis linked to proper chromosome distribution, we hypothesized that LOXL3 might be required to maintain genome stability of melanoma cells. Data mining from the TCGA [24] showed that upregulated *LOXL3* mRNA levels significantly correlate with a higher presence of copy number alterations (CNA) and overall mutations in human melanoma samples (*n* > 300 patients) (Fig. 7a, b). We then performed conventional comparative genomic hybridization (CGH) profiling of several primary and metastatic cell lines upon LOXL3 silencing, identifying many genomic aberrations associated with the loss of LOXL3 expression (Supplementary Table 3) additionally supporting LOXL3 contribution to genomic integrity in melanoma.

Finally, we explored potential mechanisms responsible for LOXL3 upregulation in melanoma tumors, in particular epigenetic control. TCGA data interrogation [24] revealed that *LOXL3* promoter methylation levels (detected with four independent probes) negatively correlate with *LOXL3* mRNA expression in melanoma tumors (Fig. 7c). Remarkably, the percentage of methylation of *LOXL3* regulatory regions inversely associates with LOXL3 expression levels in diverse melanoma cells (Fig. 7d, Fig. 1e and Supplementary Fig. 7). Moreover, upon inhibition of DNA methylation, LOXL3 is upregulated in melanoma cells with minimal endogenous LOXL3 levels (Fig. 7e). These data suggest that the hypomethylation of *LOXL3* promoter in melanoma is one factor leading to enhanced LOXL3 expression.

## Discussion

Recently, accumulating evidence has contributed to recognize the importance of lysyl oxidase family members in a plethora of biological and pathological processes ranging from ECM maturation to fibrosis, tumorigenesis and metastasis [5, 6, 8, 43]. LOX proteins have a well-established role as secreted enzymes contributing to tissue homeostasis by cross-linking ECM substrates [2, 3]. Nonetheless, the generation of independent loss-of-function mouse models of *Lox*, *Loxl1*, *Loxl2*, and *Loxl3* genes has revealed non-redundant roles and distinct contribution to tissue fitness and proper embryonic development [19–21, 44–46]. In addition to their extracellular roles, recent data support equally important intracellular functions for some LOX family members [6–9, 11]. Extensive work by our lab and others has established the deregulation of lysyl oxidases in cancer and their status has been associated with patient outcome in specific neoplasias [5, 6]. Regarding LOXL3, substantial information reinforces its role in ECM remodeling linking deregulated LOXL3 to different connective tissue disorders [16, 18–21]; however, LOXL3 involvement in cancer has not been systematically investigated. Our current *in silico* analyses in different tumor samples uncovered a clear association of LOXL3 expression to melanoma with *LOXL3* mRNA being particularly higher among melanomas carrying well-known oncogenic mutations. LOXL3 expression analyses of an ample set of human cell lines confirmed both increased mRNA and protein levels in primary and malignant melanoma samples compared to melanocytes, which were not observed for other lysyl oxidase members. Interestingly, we found that *LOXL3* hypomethylation status is associated with *LOXL3* upregulation in melanoma tumor samples and cell lines, supporting epigenetic regulation as one mechanism contributing to maintain high LOXL3 expression in this tumor type. Gain and loss-of-function studies have clarified LOXL3 involvement in melanoma and the consequences of LOXL3 deregulation in primary and metastatic melanoma cell lines. Importantly, *LOXL3* knockdown was found to be deleterious for cell proliferation in melanoma cells independently of their origin and the oncogenic mutations they harbor. LOXL3-silenced cells ultimately die through apoptosis and ectopic LOXL3 expression collaborates with oncogenic mutations such as BRAFV600E to induce transformation of immortalized human melanocytes, while forced LOXL3 expression in melanoma cells with minimal endogenous LOXL3 enhances their migration and invasion  capabilities. Accordingly, tumor growth is delayed in immunocompromised mice orthotopically injected with LOXL3-depleted melanoma cells corroborating LOXL3 involvement in melanomagenesis. Mechanistically, we propose that LOXL3 is responsible for maintaining genome stability since its depletion promotes DNA damage, partial DNA damage checkpoint activation and impaired HR activity. Moreover, LOXL3 silencing leads to a halt in cell proliferation, a delay in cell-cycle progression and defective mitotic completion. The accumulation of DSBs due to LOXL3 knockdown activate the DDR through the ATM pathway, a central player in preventing cells from entering mitosis with damaged or unreplicated DNA [47]. However, the ATR pathway is downregulated in LOXL3-depleted cells, indicating defective activation of the DDR, as demonstrated by the lower HR activity to repair DSB displayed by LOXL3-silenced cells whereas the error-prone NHEJ repair pathway remained unaffected. Importantly, LOXL3 specifically interacts with proteins required to maintain genome integrity (BRCA2, MSH2, NUMA1, SMC1A), some of which are in turn effectors of the DDR (BRCA2, MSH2, SMC1A) [36]. Noticeably, some of these LOXL3 interacting partners are downregulated upon LOXL3 depletion (BRCA2 and MSH2) as well as BRCA1 and Rad51, also required for DNA repair upon damage. Similarly, several essential effectors of mitotic entry and completion like cyclin A, cyclin B1 and AURKA, required for recovery from DNA damage checkpoint activation [48], are downregulated upon LOXL3 depletion. Moreover, LOXL3-silenced cells struggle to reach and complete mitosis, suffering mitotic catastrophe and apoptosis, or displaying mitotic abnormalities such as chromosome segregation defects and micronuclei appearance. Additionally, LOXL3 interacting protein SMC1A is involved in DSBs repair and is required throughout mitosis to ensure faithful chromosome segregation [31], whereas NUMA1 coordinates cell-cycle progression and mitotic spindle function to ensure adequate separation of DNA and cell division [33]. While NUMA1 and SMC1A protein levels remain steady upon LOXL3 silencing, we suspect their function might be compromised in the absence of LOXL3 interacting protein thus contributing to faulty mitosis and probably further enhancing DNA damage. LOXL2 interacts with and stabilizes Snail1 by hampering its ubiquitination [22]. In this line, our results suggest that LOXL3 might contribute to maintain functional levels of some of its binding partners such as BRCA2 or MSH2, whereas LOXL3-mediated regulation of NUMA1 and SMC1A function remains to be further explored. We also propose that LOXL3 roles in melanoma are mostly due to the intracellular protein, based on LOXL3 subcellular localization and its biologically relevant association to intracellular partners.

**Fig. 8:**
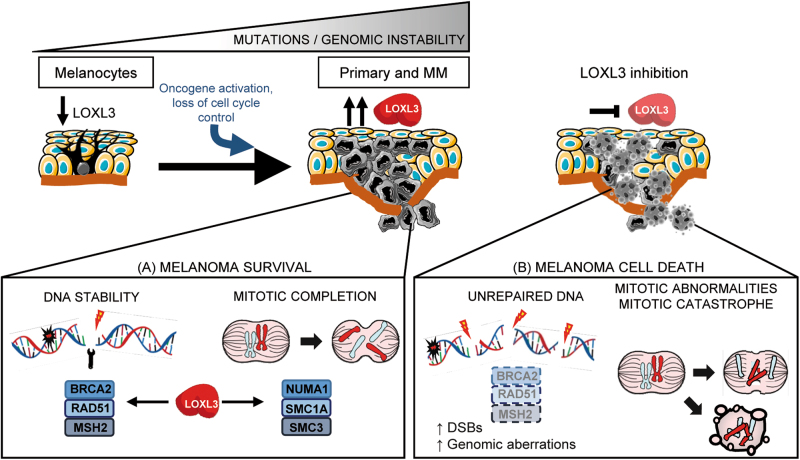
Working model depicting proposed LOXL3 roles in melanoma. Melanoma arises through the progressive accumulation of genomic alterations resulting in uncontrolled tumor cell proliferation followed by invasion and metastasis. High levels of LOXL3 **(a)** would sustain effective DNA repair and activation of cell-cycle checkpoint pathways allowing melanoma cells to deal with DNA damage. LOXL3 targeting **(b)** would hamper the activation of a competent DNA damage checkpoint leading to a halt in cell proliferation and abnormal mitosis. The increase in genomic instability due to DSBs and mitotic catastrophes would ultimately lead to apoptosis of melanoma cells

Early in tumorigenesis, melanoma cells most likely escape the DDR checkpoint to enable cell proliferation despite the accumulation of DNA damage, but later on, checkpoint-dependent cell-cycle arrest is required to prevent transformed cells from entering mitotic catastrophe [30, 49]. Melanoma is characterized by a high mutation burden [24], arising and progressing to malignancy by mechanisms still poorly understood. Our results support that the presence of LOXL3 is required for proper DDR and mitosis in melanoma cells. Upon LOXL3 silencing, melanoma cells fail to activate and execute a competent DDR required for cell survival (illustrated in Fig. 8). Besides, LOXL3 binds to proteins required for mitosis such as NUMA1 and SMC1A while LOXL3 depletion prevents proper mitotic completion. Therefore, we hypothesize that LOXL3 allows melanoma cells to survive in the presence of DNA damage since its depletion results in increased DSBs, genomic aberrations and defective mitosis leading to cell death. To our knowledge, the present data represent the first report demonstrating an involvement for LOXL3 in cancer, particularly establishing melanoma cell addiction to LOXL3, and uncover a novel association between LOXL3 and mechanisms of genome integrity maintenance.

## Materials and methods

### Public data sets analyses

Analyses regarding mRNA expression, tumor mutational status, sample origin, CNAs, overall number of mutations, and methylation status were performed at http://www.cbioportal.org/ on data of skin cutaneous melanoma (SKCM) patients generated by the TCGA Research Network: http://cancergenome.nih.gov/. LOXL3 accession number used in the analyses was uc002smp.1. For Riker et al. [25] and Raskin et al. [26] data sets, the probe of the array corresponding to LOXL3 was 228253_at.

### Cell proliferation and cell synchronization

Melanoma cell lines used in the study were obtained in 2013–2014. Hermes cell were kindly provided by D. Bennett (University of London, London, UK), most SK-MEL cell lines by A. Houghton (Memorial Sloan-Kettering Cancer Center, New York, NY), and WM cell lines (as well as 451Lu) were purchased commercially from the Wistar Institute (Philadelphia, PA). FM32 and FM35 melanocytes were kindly provided by M. Soengas, (CNIO, Madrid, Spain) and SB-Cl2 cell line by B. Jiménez (IIB, Madrid, Spain). A375P, A2058, SK-MEL-2, SK-MEL-5, SK-MEL-28, 501MEL and MeWo cells were acquired from ATCC. All experiments were performed within 5–10 passages after thawing and cells were routinely tested for mycoplasma contamination. A375P cells were authenticated by STR profiling. Melanoma cell lines culture and sources are indicated in Supplementary Data and Supplementary Table 1. For cell proliferation assays, 2500–5000 cells/well were seeded in triplicate in 24-well plates, trypsinized and counted at the indicated time intervals. For each time point, total cell number was divided by the number of cells at day 1 post-seeding. For G1/S cell synchronization assays, A375P cells were incubated with 2.5 mM thymidine (Thy, Sigma-Aldrich) for 20 h (single Thy block) or for 18 h with 2 mM Thy, washed with PBS, recovered in complete media for 6–8 h and incubated again for 18 h with 2 mM Thy (double Thy block).

### *LOXL3* interference and LOXL3 ectopic expression

LOXL3 shRNAs were obtained from the MISSION TRC shRNA human library (Sigma-Aldrich) in pLKO.1-puro vector as well as MISSION pLKO.1-puro non-mammalian shRNA (non-targeting, NTC) control plasmid (SHC002 SIGMA). shRNA scramble control (Crtl) and indicated LOXL3 shRNAs were subsequently cloned into Histone H2B-mRFP1 LV-RFP (Addgene 26001) or Histone H2B-GFP LV-GFP (Addgene 25999), respectively. LOXL3 and LOXL3Δ (lacking exons 4 and 5) FLAG-tagged cDNAs were subcloned into pWPI lentiviral vector (Addgene 12254) that allows simultaneous expression of the transgene and EGFP. Lentiviruses were packaged in HEK293T cells co-transfecting the lentiviral vector, psPAX2 (Addgene 12260) and pMD2.G (Addgene 12259) with Lipofectamine2000 DNA Transfection Reagent (Invitrogen). Melanoma cells were transduced with viral supernatant supplemented with polybrene (2 μg/mL) and either selected with 1 μg/ml puromycin for 2 days (pLKO based lentiviruses) or sorted for EGFP-positive cells using a BD FACSVantage SE cell sorter (pWPI based lentiviruses). For LOXL3 transient silencing, 100 nM of LOXL3 ON-TARGETplus SMARTpool (siSMART-L3) or control scramble siRNA (Dharmacon) were transfected in melanoma cells with Lipofectamine2000 (Invitrogen).

### Live-cell imaging analysis

For time-lapse video microscopy, A375P cells expressing Histone H2B-mRFP1 LV-RFP (Crtl) or H2B-GFP LV-GFP (shL3#1 or shL3#2) were synchronized in G1/S after incubation with 2.5 mM thymidine for 20 h and 1.5 × 10^4^ cells were seeded on camera chambers (IBIDI). Mitotic progression was followed by time-lapse microscopy starting 5 h after release in fresh media. Image acquisition was performed using a Cell Observer (Zeiss) equipped with an inverted light and fluorescence microscope and built with an environmental chamber for controlling temperature, humidity, and CO_2_. Light and fluorescence images were acquired with Plan-APOCHROMAT (40x AN1.3) objective at 5 min intervals and processed using Axiovision Rel. 4.8 software.

### Immunoprecipitation and mass spectrometry analysis

Subsequent to cell lysis, immunoprecipitation of FLAG-tagged proteins was performed using FLAG-M2 beads (Sigma-Aldrich) overnight at 4 °C. As a negative control we used cells transduced with an empty vector to hinder unspecific binding to FLAG resin. LOXL3 containing protein complexes were recovered by competitive elution with 3XFLAG peptide (Sigma-Aldrich) and analyzed by mass spectrometry. The MS/MS spectra were searched using the Uniprot human database with LOXL3 and LOXL3Δ sequences inserted using Sequest within Proteome Discoverer (Thermo Fisher). The data was filtered using a 1% false discovery rate searched against a decoy database and only proteins with at least two unique peptides were retained for further analysis. For immunoprecipitation of endogenous proteins, cell lysates were incubated with specific antibodies (Supplementary Table 4) overnight at 4 °C, followed by precipitation with protein G Dynabeads (Invitrogen) at 4 °C for 3 h.

Protocols for annexin V staining, cell-cycle analysis, soft agar, migration, and invasion assays, mouse xenografts, quantitative real-time PCR (qPCR), western blots, immunofluorescence, subcellular fractionation, CRISPR/Cas9 editing, HR and NHEJ activity, DNA methylation and array-CGH are described in the Supplementary Data.

A list of antibodies and their use is provided in Supplementary Tables 4 and 5.

### Statistical analysis

Statistical significance was performed using GraphPad Prism 6. Unless otherwise indicated, numerical data are expressed as mean ± s.e.m. Statistical significance was determined by two-sided Student’s *t*-test, one-way ANOVA, two-tailed Spearman correlation or Mann–Whitney test. All western blot analyses were independently repeated at least three times, and representative results are shown.

## Electronic supplementary material

Supplementary Information

Supplementary Figures

Supplementary Tables

Supplementary Video 1

Supplementary Video 2

Supplementary Video 3
