# Supplementary material for: Lysyl oxidase-like 3 is required for melanoma cell survival by maintaining genomic stability
Source: Cell Death Differ. 2017 Dec 11;25(5):935–50. doi: 10.1038/s41418-017-0030-2 (PMC5907912; doi:10.1038/s41418-017-0030-2)
Supplement: Supplementary file 1 — Supplementary Information [file 41418_2017_30_MOESM1_ESM.pdf]

Supplementary Data

Lysyl oxidase-like 3 is required for melanoma cell survival by maintaining genomic stability.

Authors:

Patricia G. Santamaría[1,2,6,7], Alfredo Floristán[1,6], Bárbara Fontanals-Cirera[3], Alberto Vázquez-Naharro[1,2], Vanesa Santos[1,2], Saleta Morales[1], Lourdes Yuste[1], Héctor Peinado[4], Antonio García-Gómez[5], Francisco Portillo[1,2], Eva Hernando[3] and Amparo Cano[1,2,7].

Supplementary Materials and Methods

Mouse xenografts

Control and LOXL3 silenced A375P cells were intradermically injected (1×10^6/flank) in 8-10 week old female nude mice (RjOrl:NMRI-*Foxn1*^{nu} /*Foxn1*^{nu} Janvier Labs). Tumor volume was measured once a week using a caliper and calculated with the following formula: tumor volume = $L \times W^2 \times (\pi/6)$ where L is length and W is width.

Cell culture

Melanoma cell line sources are indicated in Supplementary Table 1. Briefly, most cell lines were cultured in DMEM complete medium [10% fetal bovine serum (FBS), 20 mM glutamine, 10 U/ml penicillin and 10 µg/ml streptomycin] except for: WM cell lines (Tu2% melanoma medium, Wistar Institute, Herlyn lab); HMEL melanocytes (complete Ham´s F-10 medium) and Hermes melanocytes cultured as in[1] .

Annexin V staining and cell-cycle analysis

Lentiviral-infected cells were selected in 1 µg/ml puromycin and seeded at 0.5×10^4 cells per 6-cm dish. After 48-72 h, cells were trypsinized, washed with PBS and incubated with 0.1 µg of propidium iodide and 4 µl of Annexin-V-FITC (ANXVF-200T Immunostep) in Binding buffer (5 mM $CaCl_2$, 10 mM HEPES pH 7.4, 140 mM NaCl) and analysed in a BD FACSCanto II Flow Cytometer. For cell cycle analysis cells were fixed and permeabilized with cold ethanol 70%, washed with PBS and resuspended in propidium iodide buffer (PI 500 ng/mL, RNAse A 10 µg/mL). FACSDiVA 6.1.2 software was used to analyze the results.

Soft agar, migration and invasion assays

For anchorage independent growth assays cells were cultured in 6 cm plates previously covered in soft agar bottom layer (DMEM complete medium with 0.7% agar). The top layer contained $1x10^4$ cells in DMEM complete medium with 0.35% agar. Medium was added to the top layer to prevent drying and after 14 days, colonies were stained with 0.1% crystal violet (SIGMA-ALDRICH), photographed, and colonies bigger than 0.2 mm counted. For migration and invasion assays a suspension of $3x10^4$ melanoma cells was added to cell culture inserts (BD) containing a polycarbonate filter with 8 µm-diameter pores either non-coated (migration assays) or coated with 100 µg/mL fibronectin (invasion assays). Typically, cells were incubated for 18 h to study migration and 24 h for invasion under standard culture conditions. Cells remaining on the top-side of the membrane were removed and cells that had migrated to the underside were fixed and stained with crystal violet. Five fields per insert were photographed and scored.

Quantitative real-time PCR (qPCR)

Total RNA was extracted using RNeasy Qiagen extraction kit according to the manufacturer's instructions. One microgram of RNA was then subjected to retrotranscription. Real-time PCR amplification using SYBR green fluorescence (Applied Biosystems) and further analysis was

conducted using the iQ5 Real-Time PCR System (BioRad). β-actin or GAPDH were used as internal standards. Relative quantification of gene expression was done using the ΔΔCt method[2] . Oligonucleotide sequences are available upon request.

Western blots

Routine cell lysis was performed on ice in 50 mM Tris buffer, pH 7.5, containing: 150 mM NaCl, 0.5% Triton X-100, 50 mM NaF, 1 mM EDTA, 20 mM β-glycerophosphate and a cocktail of protease and phosphatase inhibitors. Cell lysates (50 µg of protein) were resolved in 6, 10 or 12% Tris–glycine SDS-PAGE gels and transferred to PVDF membranes (Immobilon P Millipore). Membranes were blocked for 1 h with 5% nonfat milk and probed with primary antibodies overnight at 4°C (shown in Supplementary Table 4). Membranes were incubated with horseradish peroxidase-conjugated secondary antibodies for 1 h (shown in Supplementary Table 5) before developing with chemiluminescence Western blotting substrate (Pierce).

CRISPR/Cas9 gene editing

A375P cells were transduced with Cas9 lentivirus (Addgene #52962) and a homogeneous population was selected with blasticidin (10 µg/mL). Optimized CRISPR Design tool (Zhang Lab, MIT) was used to design an sgRNA targeting exon 2 from *LOXL3* gene The sgRNA was cloned in a pLKO-GFP vector, and A375P-Cas9 cells were transduced with virus expressing a control sequence (sgSCR) or the specific sequence targeting LOXL3 (sgLOXL3). At 48 h pi, GFP+ cells were trypsinized, counted and seeded for the following experiments. Gene knockout was confirmed by genomic sequencing.

Immunofluorescence

Cells were seeded on 12 mm cover slips 24 h before the indicated time of analyses. Fixed cells were permeabilized in 0.1% Triton X-100 for 15 min. Cells were blocked with BSA 1% in PBS for 30 min at

37°C, incubated with primary antibodies for 1 h at 37°C and with secondary antibodies plus DAPI for 1 h at 37°C (Supplementary Tables 4 and 5). Coverslips were mounted on microscope slides with FluorPreserve Reagent (Calbiochem). Images were taken with the automated microscope Nikon90i and foci analysis was performed using ImageJ program. Confocal images were acquired on a Zeiss LSM 710 confocal microscope equipped with a Axiocam MRm (B/W) CCD camera using a 63x NA 1.0 oil objective and processed in Zeiss software maintaining relative image intensities. For co-localization analyses (BRCA2 and LOXL3) the Manders' coefficients were derived with the JACoP ImageJ tool (http://rsb.info.nih.gov/ij/plugins/track/jacop.html) and are the average of 250 individual cells. Details for antibodies dilutions and fixation methods are provided in Supplementary Table 4.

Cell fractionation

Subcellular fractionation of A375P cells was either performed using a commercial kit (Thermo Scientific, #78840) or by differential centrifugation. Briefly, pellets from indicated cells were re-suspended in HEPES-Sucrose Buffer (HBS) (0.25 M sucrose, 10 mM HEPES pH 7.4, 1 mM EDTA and 1mM DTT) and lysed with 30 strokes using a tight homogenizer. Samples were centrifuged at 100 g for 2 min at 4°C and the supernatant was further centrifuged at 600 g for 10 min at 4°C to obtain a pellet or nuclear fraction (P600) and a cytosolic fraction (S600). S600 was further centrifuged at 160,000 g for 1 h at 4°C (TL-A100 3 rotor, Optima TLX Ultracentrifuge, Beckman). The resulting supernatant is enriched in the cytosolic soluble fraction (S160) while the pellet (P160) in subcellular organelles. For nuclear subfractionation, P600 was lysed in BLN buffer (20 mM HEPES pH 7.5, 150 mM NaCl, 1.5 mM $MgCl_2$, 0.5 mM DTT and 0.6% Triton X-100) for 5 min at 4°C. The sample was then centrifuged at 16,000 g for 5 min at 4°C in a microfuge to obtain the soluble nuclear fraction (S20) and the bound chromatin fraction (P20). P20 was sonicated (18 cycles for 30 s) and further

centrifuged at 16,000 g for 5 min at 4°C to solubilize the chromatin bound proteins (Ssonic). Enrichment of the different subcellular compartments was analyzed by western blot, using the appropriate controls (GM130, Lamin A/C, Histone 3 and α-tubulin) for each fraction.

Homologous recombination activity

The pHPRT-DRGFP reporter consists of two defective GFP genes, the first of which contains an I-SceI endonuclease site such that transduced cells are GFP negative (Figure 5i). Cellular expression of I-SceI leads to a DSB which can be repaired by HR using the downstream wild-type GFP sequence as a template, resulting in GFP positive cells[3] . The reporter vector pHPRT-DRGFP (Addgene#26476, a gift from M. Malumbres) was transfected into A375P cells and stable clones were obtained after selection with 1 µg/mL puromycin. A375P pHPRT-DRGFP cells were thus transduced with control (NTC) and shLOXL3 lentivirus (shL3#1 and shL3#2). 24 h pi, cells were transfected with pCBA-SceI (Addgene#26477), a plasmid encoding the I-SceI endonuclease, which targets the pHPRT-DRGFP vector generating a DSB. The successful repair of the DSB through homologous recombination (HR) restores the GFP gene sequence, turning the cell into GFP positive. A vector expressing NLS-Cherry was transfected as an internal control of transfection efficiency. At 72 h post-transfection cells were analyzed for GFP and Cherry using a Beckman Coulter FC500 cytometer.

Non homologous end joining activity

The NHEJ-GFP reporter plasmid (a gift from M. Malumbres) harbors an inactive GFP gene with an artificially engineered intron, interrupted by an adenoviral exon, flanked by restriction sites for induction of DSBs. Upon digestion with *Hind*III or I-SceI enzymes and transfection into cells, successful NHEJ events turn the cells GFP+[4] .

A375P cells were infected with control (NTC) and shLOXL3 lentivirus (shL3#1 and shL3#2). 24 h pi, cells were co-transfected with different amounts of *Hind*III digested NHEJ-GFP reporter (0-2 µg) and a vector expressing NLS-Cherry (internal control for transfection efficiency). At 48 h post-transfection cells were analyzed for GFP and Cherry using a Beckman Coulter FC500 cytometer.

Array CGH

Array-CGH analyses[5] were conducted on oligonucleotide-based SurePrint G3 Human CGH 8×60K microarray slides that have a backbone resolution of ~240 Kb (Agilent Technologies, Santa Clara, CA, USA). Sample DNAs (from shL3#1 and shL3#2 infected cells) were labeled with Cy5 dye and the NTC DNA samples from each cell line were used as reference DNA and labeled with Cy3 dye. Hybridizations were performed according to the manufacturer's protocols. Arrays were scanned using the G2565BA DNA Microarray Scanner (Agilent Technologies), data were extracted using the Feature Extraction Software v10.7 (Agilent Technologies) and analyzed using the Agilent Genomic Workbench v7.0 (Agilent Technologies). Aberrant regions were recognized using ADM-2 algorithm with threshold set to 6.0. Amplifications and deletions were defined if at least three consecutive probes were simultaneously changed with a minimum Average Absolute Log Ratio of 0.25.

DNA methylation analysis by pyrosequencing

DNA samples from the indicated melanoma cell lines were bisulfite-converted using the EZ DNA methylation kit (Zymo Research, Orange, CA, USA). Briefly, 2 µl of the converted DNA (corresponding to approximately 20–30 ng) were used as a template in each subsequent PCR. Primers for PCR amplification and sequencing were designed with the PyroMark® Assay Design 2.0 software (Qiagen GmbH, Hilden, Germany). PCRs were performed with the HotStart Taq DNA polymerase PCR kit (Qiagen), and PCR product amplification was assessed by agarose gel electrophoresis. PCR products

were pyrosequenced with the Pyromark Q24 system (Qiagen). Results from bisulfite pyrosequencing are presented as percentage of methylation.

References to Supplementary Materials and Methods

1. Sviderskaya EV, Gray-Schopfer VC, Hill SP, Smit NP, Evans-Whipp TJ, Bond J *et al*. p16/cyclin-dependent kinase inhibitor 2A deficiency in human melanocyte senescence, apoptosis, and immortalization: possible implications for melanoma progression. *J Natl Cancer Inst* 2003; **95**: 723-732.

2. Livak KJ, Schmittgen TD. Analysis of relative gene expression data using real-time quantitative PCR and the 2(-Delta Delta C(T)) Method. *Methods* 2001; **25**: 402-408.

3. Pierce AJ, Hu P, Han M, Ellis N, Jasin M. Ku DNA end-binding protein modulates homologous repair of double-strand breaks in mammalian cells. *Genes Dev* 2001; **15**: 3237-3242.

4. Seluanov A, Mittelman D, Pereira-Smith OM, Wilson JH, Gorbunova V. DNA end joining becomes less efficient and more error-prone during cellular senescence. *Proc Natl Acad Sci USA* 2004; **101**: 7624-7629.

5. Pinkel D, Albertson DG. Array comparative genomic hybridization and its applications in cancer. *Nat Genet* 2005; **37** Suppl:S11-7.

References to Supplementary Table 1

6. Garraway LA, Widlund HR, Rubin MA, Getz G, Berger AJ, Ramaswamy S *et al*. Integrative genomic analyses identify MITF as a lineage survival oncogene amplified in malignant melanoma. *Nature* 2005; **436**: 117-122.

7. Kaplan FM, Shao Y, Mayberry MM, Aplin AE. Hyperactivation of MEK-ERK1/2 signaling and resistance to apoptosis induced by the oncogenic B-RAF inhibitor, PLX4720, in mutant N-RAS melanoma cells. *Oncogene* 2011; **30**: 366-371.

8. Matin RN, Chikh A, Chong SL, Mesher D, Graf M, Sanza P *et al*. p63 is an alternative p53 repressor in melanoma that confers chemoresistance and a poor prognosis. *J Exp Med* 2013; **210**: 581-603.

9. Arozarena I, Goicoechea I, Erice O, Ferguson J, Margison GP, Wellbrock, C. Differential chemosensitivity to antifolate drugs between RAS and BRAF melanoma cells. *Mol Cancer* 2014; **13**: 154.

10. Xing F, Persaud Y, Pratilas CA, Taylor BS, Janakiraman M, She QB *et al*. Concurrent loss of the PTEN and RB1 tumor suppressors attenuates RAF dependence in melanomas harboring (V600E)BRAF. *Oncogene* 2012; **31**: 446-457.

11. Zhuang D, Mannava S, Grachtchouk V, Tang WH, Patil S, Wawrzyniak JA *et al*. C-MYC overexpression is required for continuous suppression of oncogene-induced senescence in melanoma cells. *Oncogene* 2008; **27**: 6623-6634.

12. Halaban R, Zhang W, Bacchiocchi A, Cheng E, Parisi F, Ariyan S *et al*. PLX4032, a selective BRAF(V600E) kinase inhibitor, activates the ERK pathway and enhances cell migration and proliferation of BRAF melanoma cells. *Pigment Cell Melanoma Res* 2010; **23**: 190-200.

13. Gao L, Feng Y, Bowers R, Becker-Hapak M, Gardner J, Council L *et al*. Ras-associated protein-1 regulates extracellular signal-regulated kinase activation and migration in melanoma cells: two processes important to melanoma tumorigenesis and metastasis. *Cancer Res* 2006; **66**: 7880-7888.

14. Ranzani M, Alifrangis C, Perna D, Dutton-Regester K, Pritchard A, Wong K *et al*. BRAF/NRAS wild-type melanoma, NF1 status and sensitivity to trametinib. *Pigment Cell Melanoma Res* 2015; **28**: 117-119.

15. Wajapeyee N, Serra RW, Zhu X, Mahalingam M, Green MR. Oncogenic BRAF induces senescence and apoptosis through pathways mediated by the secreted protein IGFBP7. *Cell* 2008; **132**: 363-374.

Supplementary Figure Legends

Supplementary Figure 1.

LOXL3 expression in human melanoma. (**a**) *LOXL3* normalized mRNA levels determined by The Cancer Genome Atlas (TCGA) in primary and metastatic human melanoma samples from a large sample cohort (n=330 patients). Error bars represent sem. ns: not statistically significant. (**b**) Characterization of LOXL3 expressing isoforms in the indicated human melanoma cell lines by RT-PCR from total isolated RNA. Main PCR products amplified (indicated by arrows) were sequenced and identified as full length *LOXL3* and *LOXL3ΔE4E5*, respectively. Asterisk (*) indicates an intermediate PCR product that could not be isolated and further characterized. (**c**) Diagram depicting LOXL3 protein domain arrangement and the differential oligonucleotide sequence obtained upon analysis corresponding to full length LOXL3 (top) and the splicing variant LOXL3 ΔE4E5 (bottom) lacking exons 4 and 5 corresponding to scavenger receptor cysteine rich 2 domain (SRCR2).

Supplementary Figure 2.

LOXL3 depletion in human melanoma cells. (**a**) LOXL3, LOXL3Δ, LOXL2 and LOX protein levels analyzed by western blot at 32 and 56 h post-infection (h pi) in A375P control (NTC) and LOXL3-silenced cells (shL3#1 and shL3#2). (**b**) SK-MEL-28 melanoma cells were either transduced with non-targeting control (NTC) and LOXL3 targeting shRNAs (shL3#1 and shL3#2) and selected with 1 µg/ml of puromycin for 48 h, or transiently transfected with control siRNA (siC) or a smart pool of siRNAs against LOXL3 (siSMART-L3) and collected after 48 h. Left, protein extracts were analyzed by immunoblot with LOXL3 antibody to confirm LOXL3 silencing. LOXL3 and LOXL3Δ isoforms are indicated. Cleaved caspase-3 was also analyzed. Right, percentage of apoptotic cells in SK-MEL-28 cell line infected or transfected as in (b) and determined by flow cytometry analysis of annexin V

binding and propidium iodide (PI) uptake. The percentage of cells annexin V positive and double positive for both annexin V and PI is indicated. One out of two representative experiments is shown. (**c**) A375P-Cas9 cell line transduced with pLKO vector expressing a control sequence (sgSCR) or a specific sequence targeting exon 2 from *LOXL3* (sgLOXL3). Cells were infected and selected with 2 µg/ml of puromycin for two days before LOXL3 protein levels were analyzed by western blot, as well as the cleavage of caspase-3. (**d**) A375P-Cas9 cells were infected and selected as in (c) before cell proliferation (left) was analyzed. A representative cell proliferation curve is shown with four experimental replicates for each time point; error bars represent sd. n=3 biologically independent replicates. Right, cells were infected as in (c) and attached and floating cells were collected 5 dpi before annexin V/PI analyses by FACS. A representative experiment out of 3 is shown. (**e**) Sequencing of genomic DNA from A375P-Cas9 cells transduced with sgSCR and sgLOXL3. gDNA was extracted at 3 dpi and 100 ng were used to amplify exon 2 from *LOXL3*. The region targeted by sgLOXL3 is indicated. The noisy signal in A375P-Cas9 sgLOXL3 sequence displays the heterogeneity in genomic DNA upon Cas9 endonuclease activity and cellular repair, which generates insertions/deletions. α-tubulin was used as a loading control (a-c). Molecular weight (MW) of protein standards (kDa) is indicated.

Supplementary Figure 3

LOXL3 promotes migration and invasion in metastatic human melanoma cells. (**a**) 501MEL metastatic melanoma cells were infected with GFP expressing lentivirus control (EV), FLAG-LOXL3 or FLAG-LOXL3ΔE4E5 isoform (LOXL3Δ) and GFP-positive sorted cells were analyzed for the expression of LOXL3 isoforms by western blot with anti-FLAG antibody. β-actin was used as a loading control. (**b**) Cell proliferation analysis of indicated infected cells as in (a) with three experimental replicates is shown. Error bars represent sem. The experiment was repeated twice with similar

results. (**c** and **d**) 501MEL cells infected as in (a) were subjected to migration (**c**) and invasion (**d**) assays. Graphs depict the number of migrating and invading cells quantified for each cell line. n=3 biologically independent replicates. (**e**) Cell proliferation of 501MEL cells transduced with lentiviral particles expressing control (NTC) and two different LOXL3 targeting shRNAs (shL3#1 and shL3#2). Cells were infected and selected with 1 µg/ml of puromycin for two days before cell proliferation was analyzed. Time points in the x-axis indicate days post-infection (dpi). (**f**) Downregulation of LOXL3 protein levels from cells infected as in (e) was verified by western blot, as well as the cleavage of caspase-3, indicative of apoptosis induction. α-tubulin was used as a loading control. (**g**) Percentage of apoptotic cells from cells infected as in (e) as determined by detection of annexin V binding and propidium iodide (PI) uptake by FACS. Representative blots and flow cytometry experiments are shown. Error bars represent sem. ∗$p<0.05$, ∗∗$p<0.01$ by a two-sided Student's t-test. MW of protein standards (kDa) is indicated.

Supplementary Figure 4.

LOXL3 co-immunoprecipitation and subcellular localization. (**a**) Endogenous LOXL3 was immunoprecipitated from A375P whole cell lysates and the binding of SMC1A to endogenous LOXL3 was confirmed by western blot. (**b**) A375P cells were subjected to cell fractionation and major subcellular fractions were analyzed by immunodetection of specific proteins associated to each cellular compartment: α-tubulin in cytoplasm, EGFR and calnexin in cell and organelle membranes, respectively; Snail1 in cell nuclei and cytoplasm; and trimethylated Histone 3 (H3 trimet) in cell nuclei and chromatin fraction. The extracellular fraction corresponds to A375P conditioned medium. (**c**) Subcellular fractions obtained by differential centrifugation of A375P and A375P-LOXL3 (transduced with FLAG-LOXL3) cell lysates were subjected to western blot analyses to determine the presence of LOXL3 and its identified binding partners (BRCA2, NUMA1, SMC1A and MSH2) in

cytoplasmic and nuclear enriched fractions. Immunodetection of different proteins was used as a control of subcellular fractionation as detailed in Supplementary Materials and Methods: GM130 and α-tubulin (cytosolic enriched fractions: S600, S160, P160), Lamin A/C and Histone 3 (H3) (nuclear enriched fractions: P600, S20, Ssonic).

Supplementary Figure 5.

DNA damage analyses in A375P cells. Quantitation of 53BP1 (**a**) and γH2AX (**b**) immunofluorescence foci upon DNA damage induction with 2 mM hydroxyurea (HU) (a) or upon ionizing radiation (IR) (b). A375P cell were incubated in the presence of HU or irradiated with 1 Gy of IR. Treated and non-treated cells were cultured for the indicated times, fixed and processed for immunofluorescence. In parallel, A375P cells were transduced with control (NTC) and LOXL3 targeting (shL3#2) lentivirus and collected at 56 h pi, fixed and processed for 53BP1 (a) and γH2AX (b). Error bars represent sem, n=2 biologically independent replicates (200 total cells were analyzed in each condition). $*p<0.05$, $**p<0.01$ by a two-sided Student's t-test. (**c**) Whole protein extracts from control (NTC) and LOXL3-silenced (shL3#2) A375P cells after 32 and 56 h pi were subjected to western blot analyses with the indicated antibodies. One out of two experiments is shown. (**d**) A375P cells were incubated in the presence of HU or irradiated with 1 Gy of IR, collected at the indicated times and whole protein extracts were analyzed by western blot with the indicated antibodies. One representative experiment (n=3) is shown. (**e**) Protein levels of crucial mediators of homologous recombination (HR) in LOXL3-silenced cells. A375P pHPRT-DRGFP cells were transduced with control (NTC) and shLOXL3 lentivirus (shL3#1 and shL3#2). 24 h pi, cells were transfected with pCBA-I-SceI plasmid and NLS-Cherry vector as a control of transfection efficiency (detailed in Supplementary Materials and Methods). 24 h later cells were collected and western blot analyses were performed on whole cell lysates to detect BRCA1, BRCA2, Rad51 and LOXL3 protein levels. α-tubulin was used as a loading

control (c-e) MW from protein standards (kDa) is indicated. (**f**) NHEJ activity represented as the percentage of GFP positive cells analyzed by FACS from A375P cells transduced with control (NTC) and LOXL3 shRNA lentivirus (shL3#1 and shL3#2) and then transfected with different amounts of linearized NHEJ-GFP reporter plasmid. n=2 biologically independent experiments.

Supplementary Figure 6.

LOXL3 silencing affects cell cycle progression. (**a**) Percentage of control (NTC) and LOXL3-silenced (shL3#1 and shL3#2) A375P cells in G0/G1, S, G2/M and sub-G1 phases, as determined by FACS analysis of DNA content at 56 and 80 h post-infection (h pi). Graph shows representative results from 3 independent experiments. (**b**) A375P cells transduced with non-targeting (NTC) and LOXL3 silencing (shL3#1 and shL3#2) lentivirus were synchronized in G1/S with double thymidine block and their cell cycle progression was followed by FACS upon release from G1/S in fresh medium. Graph depicts the percentage of cells that are in G2/M phase at the indicated time points after thymidine release. A representative experiment out of three independent replicates is shown. (**c**) Quantitation of the percentage of control (NTC) and LOXL3-silenced cells (shL3#1 and shL3#2) from infected cells on (b) at 11 and 13 h after release from the double thymidine block. Error bars represent sem. $*p<0.05$, $***p<0.001$ by a two-sided Student's t-test. (**d**) A375P cells synchronized by serum starvation for 48 h and released into fresh medium were collected at different time points and their cell cycle profile was analyzed by FACS (upper panels), and protein extracts were immunoblotted with LOXL3 antibody (bottom panels). LOXL3 and LOXL3Δ isoforms are indicated. α-tubulin was used as a loading control. (**e**) A375P cells transduced with control and LOXL3 targeting (shL3#1 and shL3#2) lentivirus were synchronized in G1/S with 2.5 mM thymidine for 20 h and their mitotic progression was followed by high-resolution fluorescence videomicroscopy and automated image analysis (Figure 6). The table shows the percentage of cells that having entered mitosis after

thymidine release die by mitotic catastrophe. The total number of analyzed cells and percentage of mitotic catastrophes in each cell culture is indicated.

Supplementary Figure 7.

Scheme depicting the CpG loci in *LOXL3* intron 1 analyzed by pyrosequencing in different melanoma cell lines. Main CpGs (marked in bold blue or bold orange and underlined) were analyzed with the indicated (TCGA). Additional probes were designed to analyze closer CpGs (marked in blue light, blue green and orange light) as indicated (CpG 1-3).

Supplementary Tables

Table 1. List of human cell lines used in the present study.

Human primary and immortalized melanocytes and melanoma cell lines used in this study depicting their origin, *NRAS/BRAF* mutational status, source and corresponding reference. NA: not analyzed/annotated. *Cell lines used for the analysis by qPCR of *LOXL3* mRNA expression (Figure 1e). RGP: radial growth phase, VPG: vertical growth phase.

Table 2. List of common proteins identified by mass spectrometry related to DDR and mitosis in 501MEL cells overexpressing LOXL3 or LOXL3Δ. The number of peptide spectrum matches (PSM) for each protein is indicated.

Table 3. List of genomic alterations found by array-CGH analyses in different cell lines upon LOXL3 silencing.

Control and LOXL3-silenced cells were collected at 72 h pi and analyzed by array-CGH using NTC as control cells. For A375P cells, two biologically independent experiments were done (I) and (II). In

the latter, two different time points post-infection were analyzed: 72 h and 96 h. CHR: chromosome. For the rest of the cell lines, analyses were done at 72 h.

Table 4. List of primary antibodies used for western blot, immunofluorescence and immunoprecipitation analyses. Dilution and fixation conditions are indicated.

Table 5. List of secondary antibodies used for western blot and immunofluorescence analyses.

Supplementary videos. Time-lapse imaging of mitotic progression of control (Supplementary video V1) and LOXL3-silenced A375P cells (Supplementary videos V2 and V3) upon G1/S synchronization and release.
