# Supplementary material for: Lysyl oxidase-like 3 is required for melanoma cell survival by maintaining genomic stability
Source: Cell Death Differ. 2017 Dec 11;25(5):935–50. doi: 10.1038/s41418-017-0030-2 (PMC5907912; doi:10.1038/s41418-017-0030-2)

Supplementary Figure 1

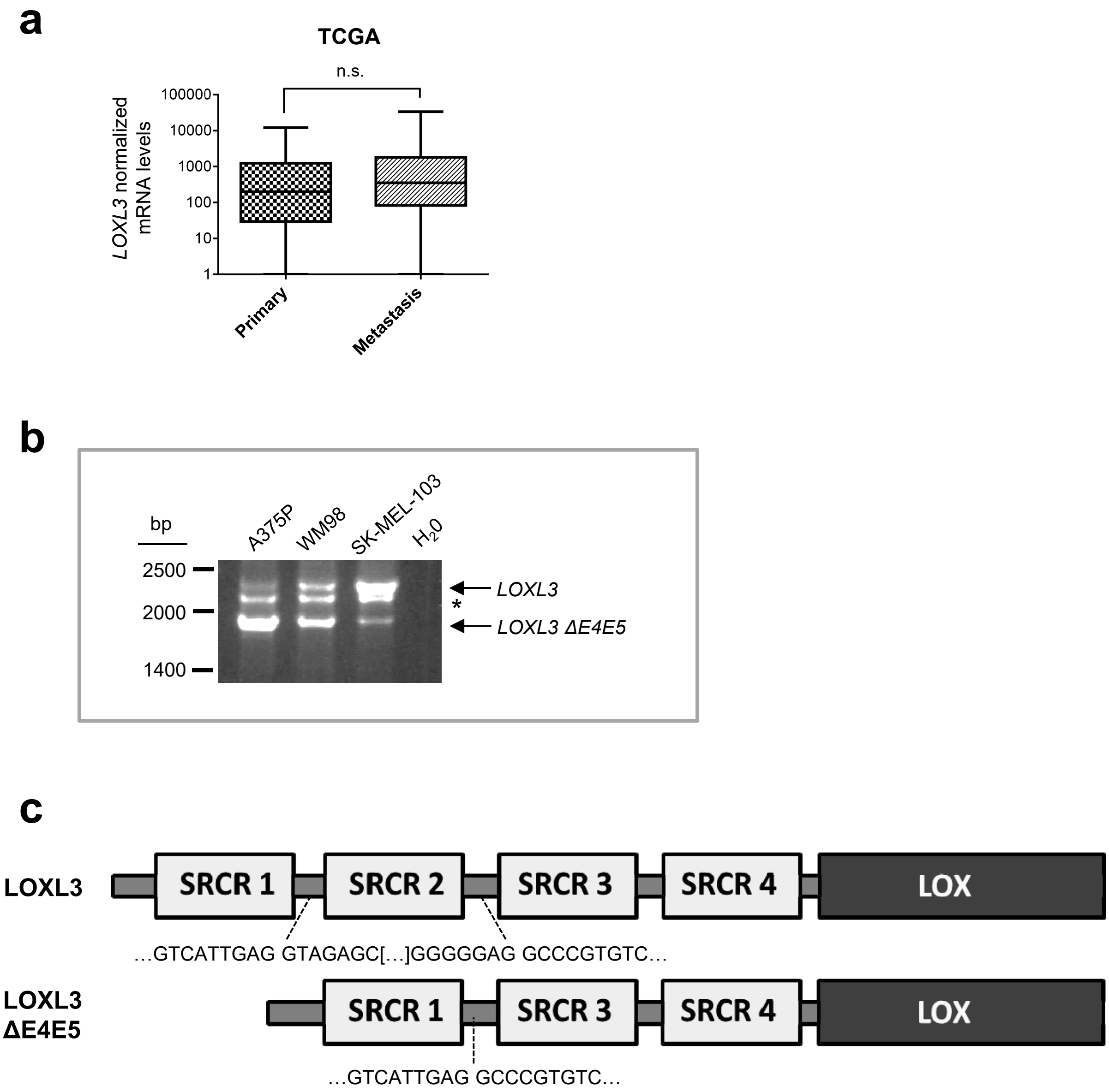

Supplementary Figure 2

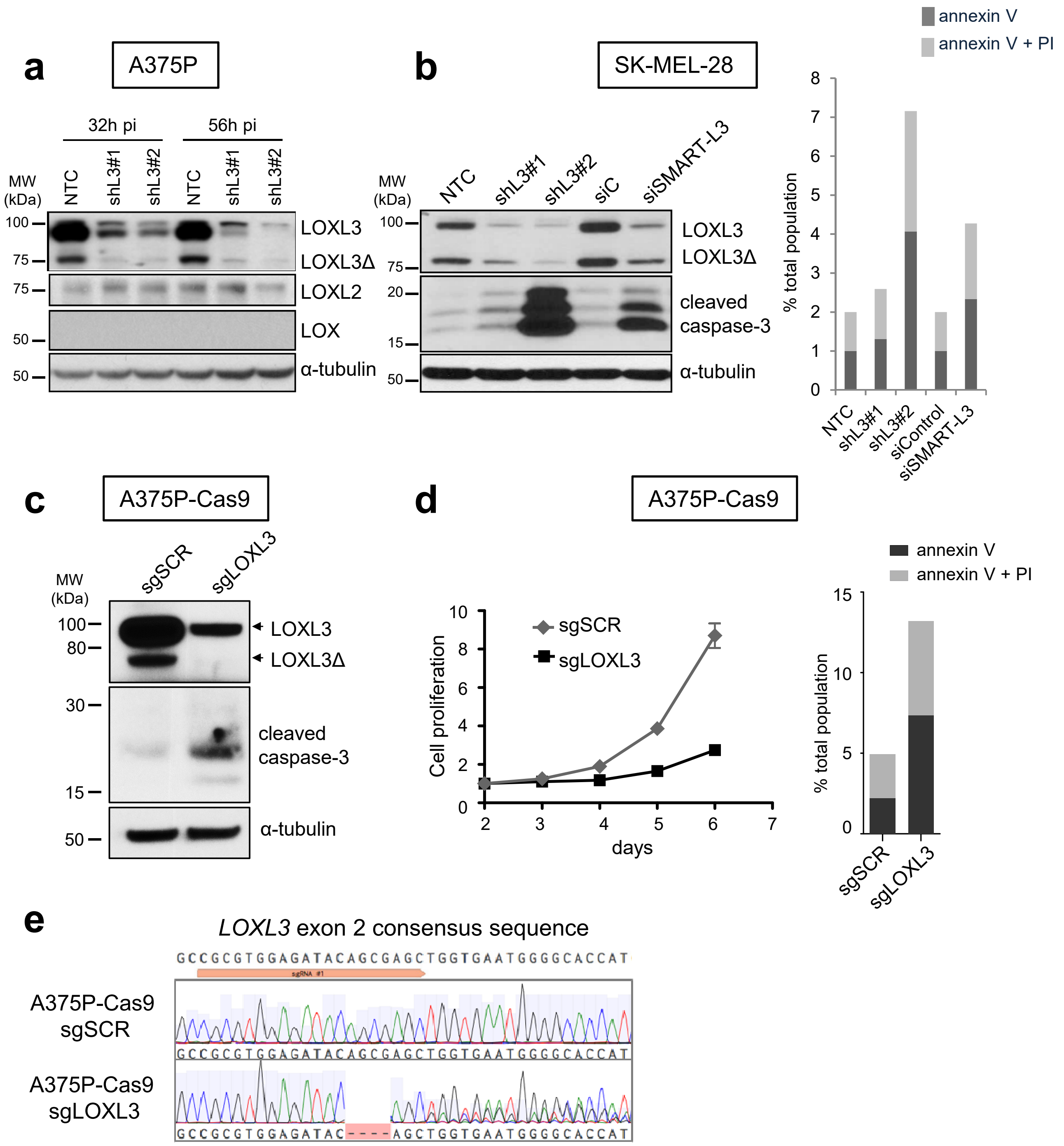

Supplementary Figure 3

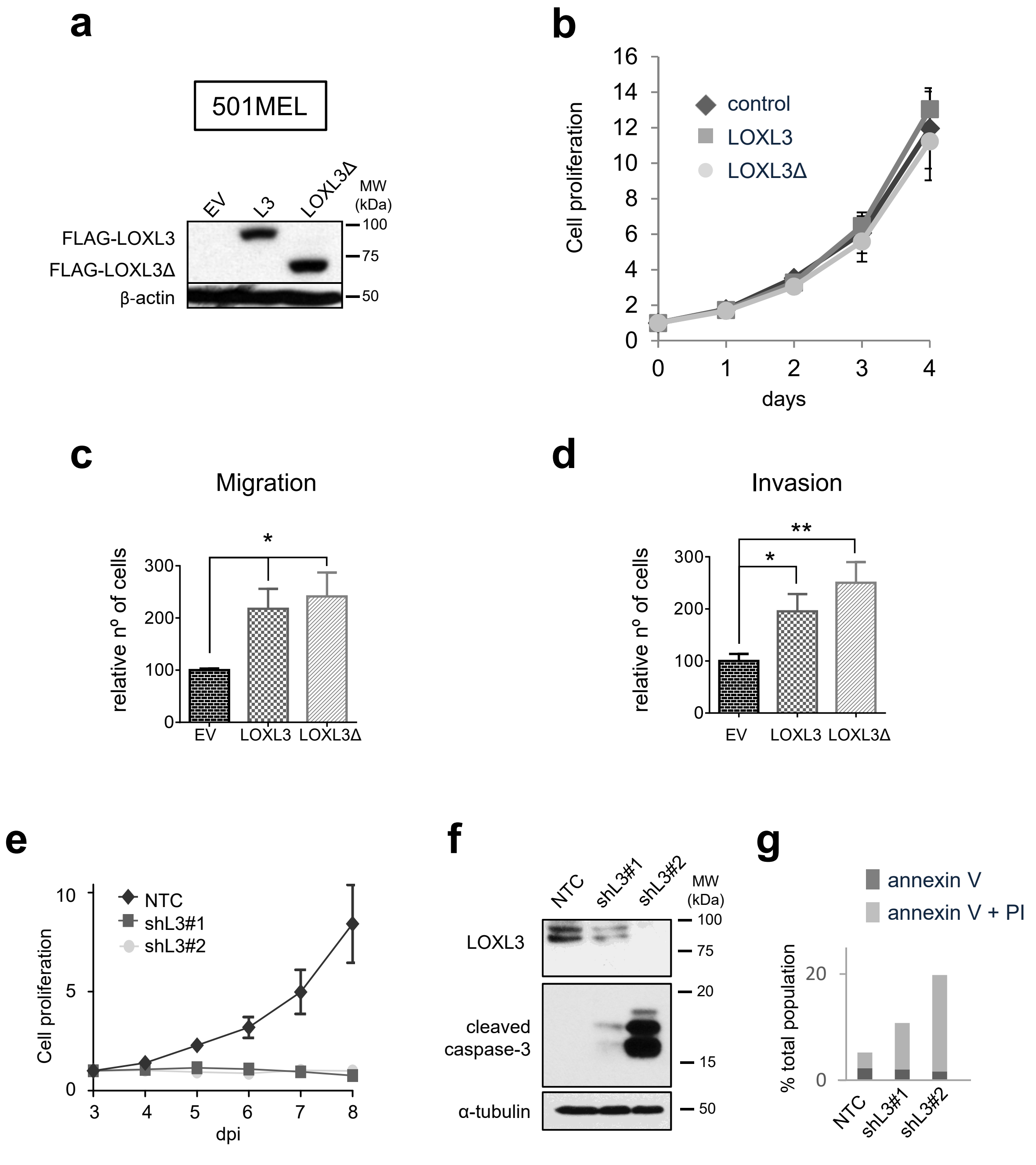

Supplementary Figure 4

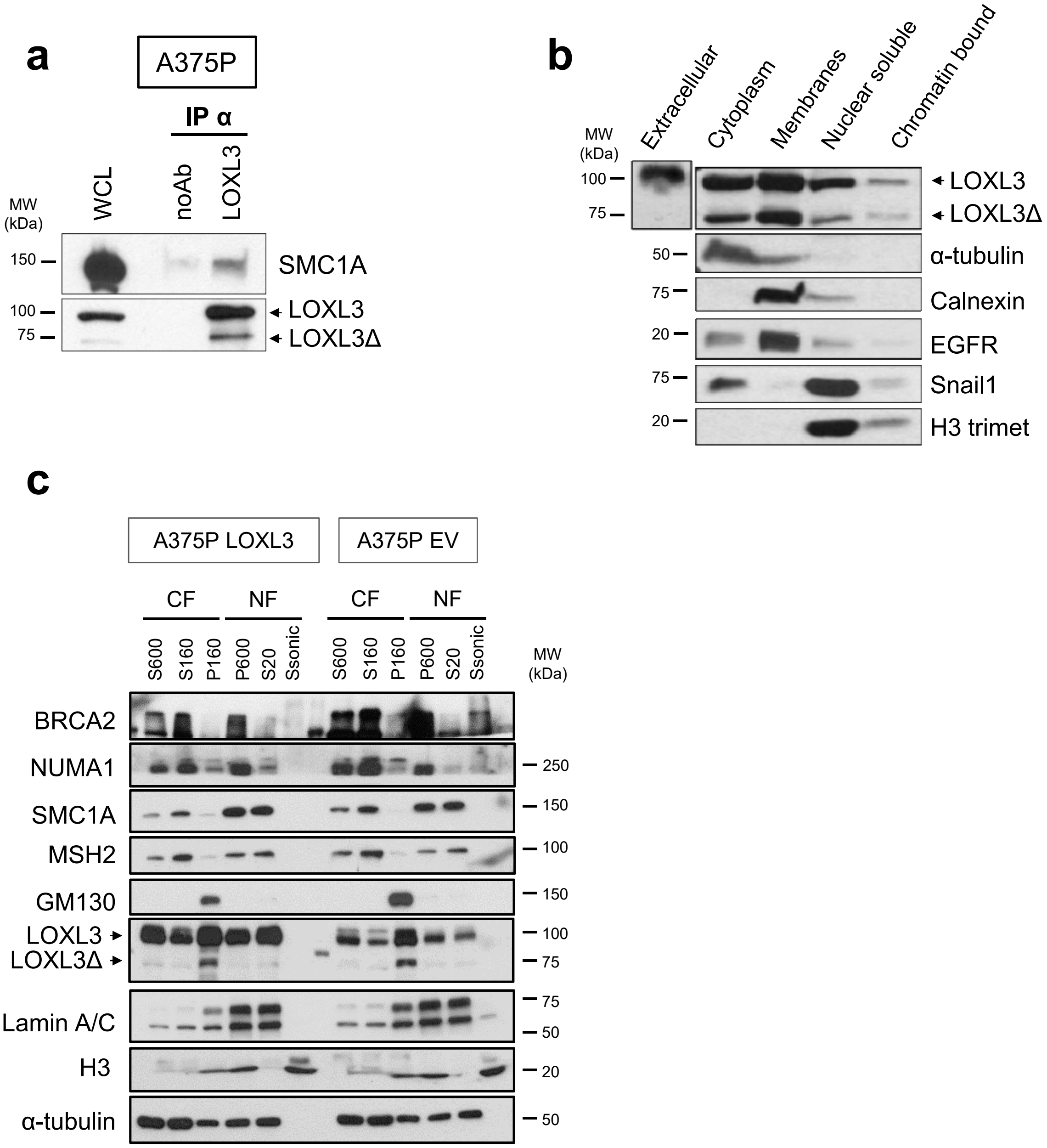

Supplementary Figure 5

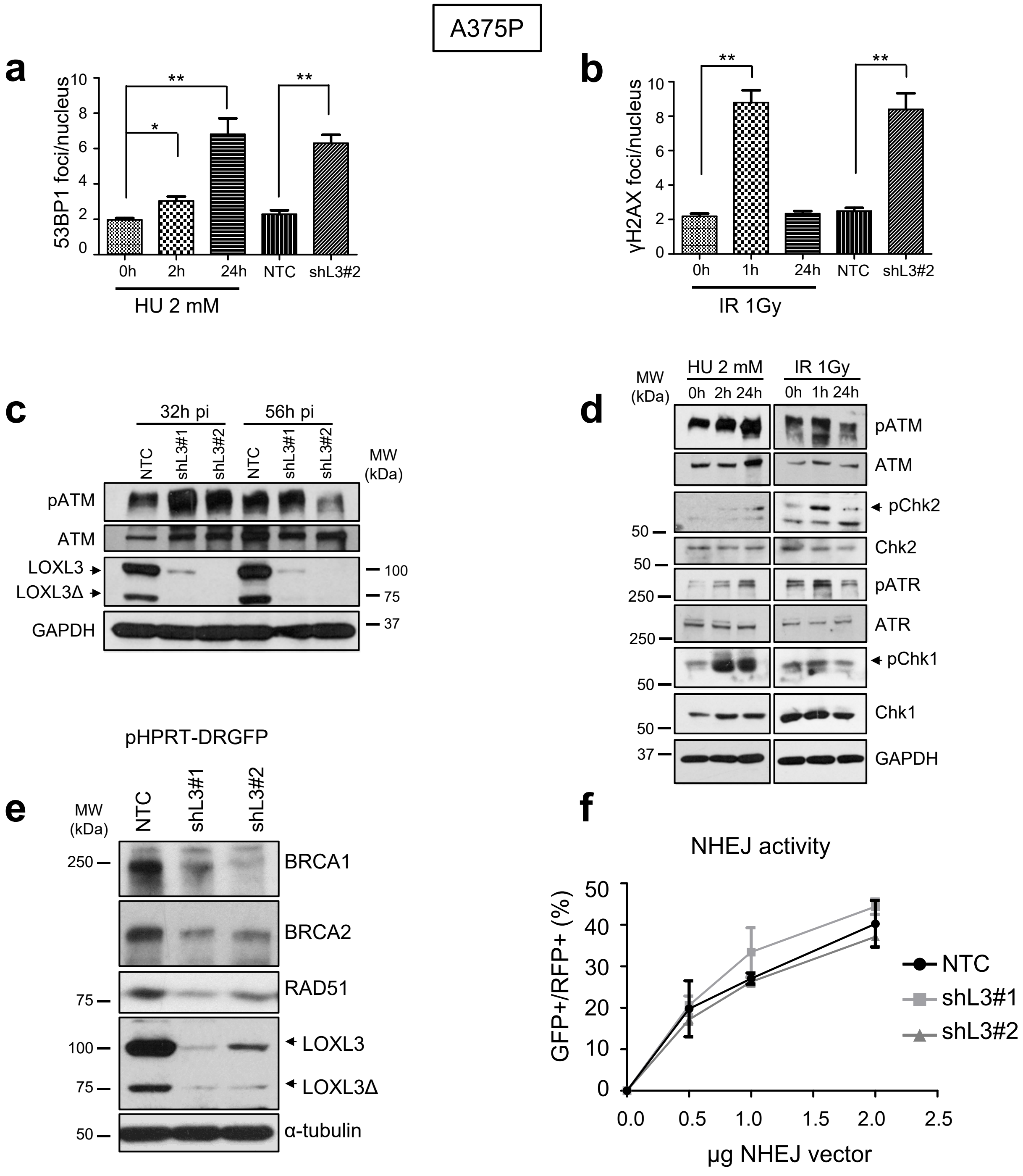

Supplementary Figure 6

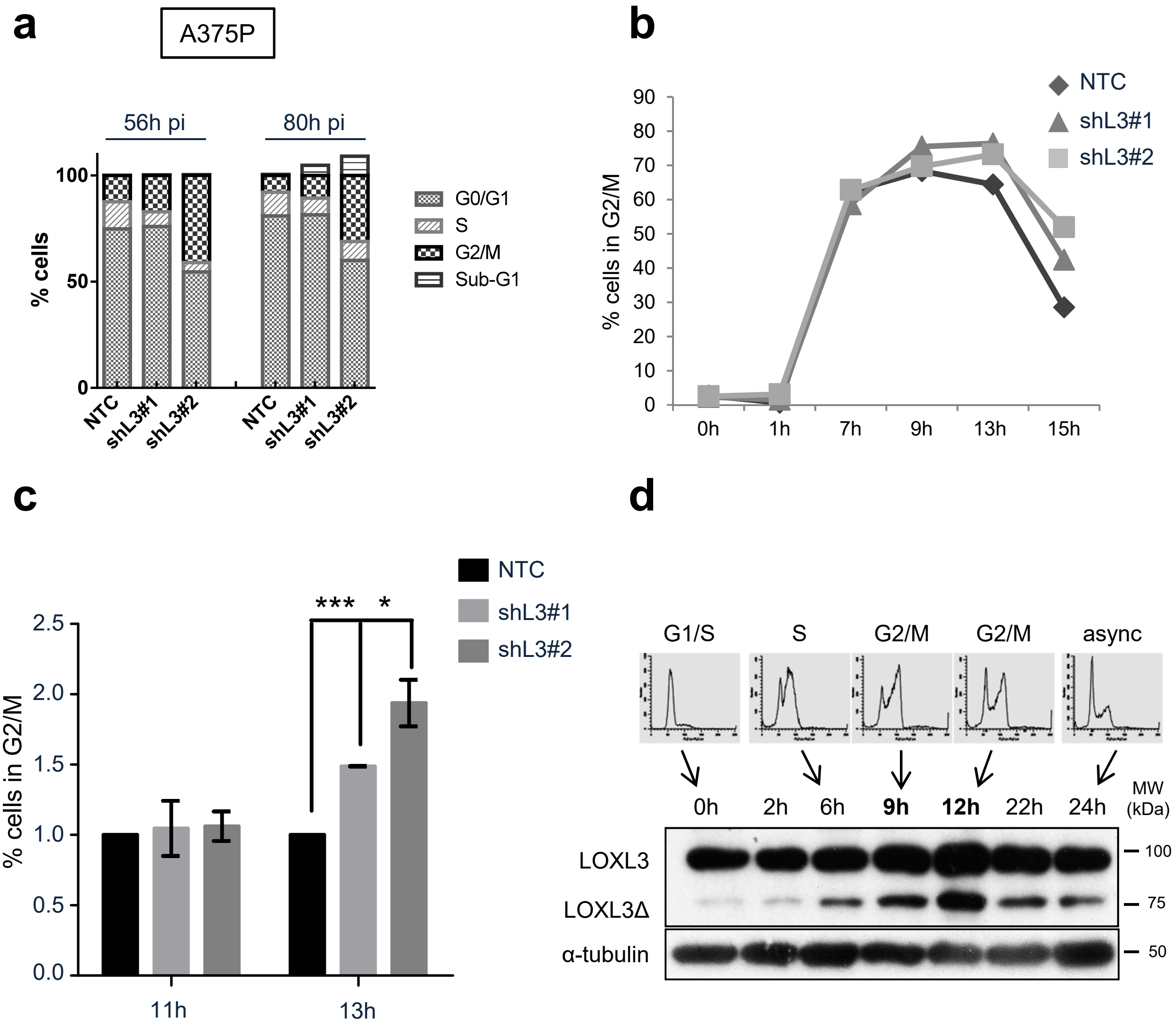

e

Mitotic catastrophes

Ctrl	1/186	(0.5%)
shL3#1	9/108	(8.3%)
shL3#2	5/50	(10%)

Supplementary Figure 7

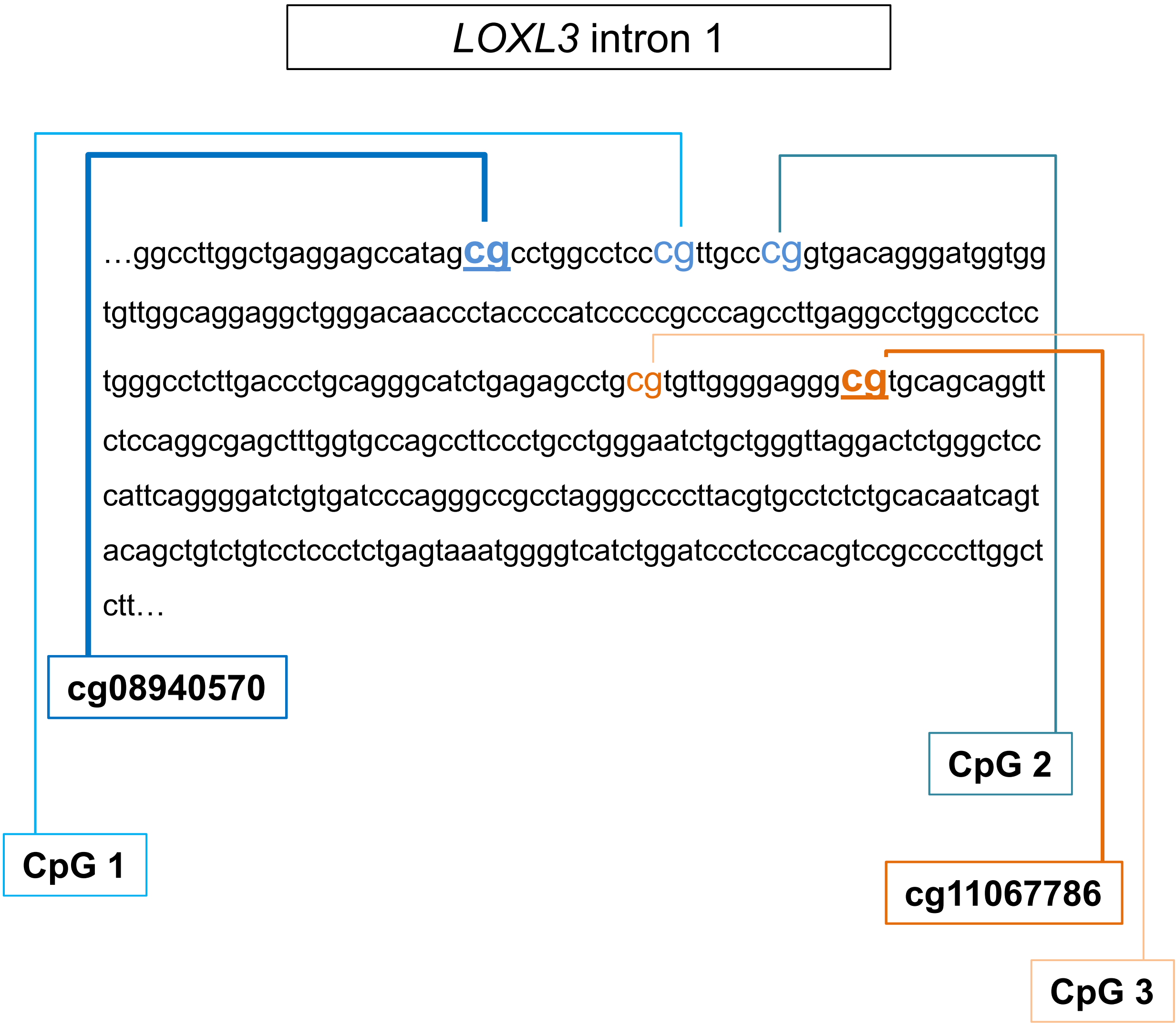

Supplement: Supplementary file 2 — Supplementary Figures [file 41418_2017_30_MOESM2_ESM.pdf]
