# Supplementary material for: Lysyl oxidase-like 3 is required for melanoma cell survival by maintaining genomic stability
Source: Cell Death Differ. 2017 Dec 11;25(5):935–50. doi: 10.1038/s41418-017-0030-2 (PMC5907912; doi:10.1038/s41418-017-0030-2)
Supplement: Supplementary file 3 — Supplementary Tables [file 41418_2017_30_MOESM3_ESM.pdf]

Supplementary Table 1

Table 1. List of cell lines used in this study

Cell line	Origin and/or disease status	NRAS/BRAF status	Source and/or reference
NHM*	Human melanocytes	NA	Hernando lab
FM32	Human melanocytes	NA	Soengas lab
FM35	Human melanocytes	NA	Soengas lab
Hermes 1*	Immortalized melanocytes	NA	Hernando lab
Hermes 2B*	Immortalized melanocytes	NA	Hernando lab
PMEL*	Immortalized melanocytes	WT	Hernando lab[6]
HMEL*	Immortalized melanocytes	BRAF V600E	Hernando lab[6]
SB-Cl2	Primary melanoma, RGP	NRAS Q61R	B. Jiménez[7]
WM35*	Primary melanoma, RGP	BRAF V600E	Wistar Institute[8]
WM1575*	Primary melanoma, RGP	BRAF V600E	Wistar Institute[8]
WM902b*	Primary melanoma, VGP	BRAF V600E	Herlyn lab, Wistar Institute
WM3211*	Primary melanoma, VGP	WT	Herlyn lab, Wistar Institute
WM98-1*	Primary melanoma, VGP	BRAF V600E	Wistar Institute[9]
WM115	Primary melanoma, VGP	BRAF V600D	Herlyn lab, Wistar Institute
WM278*	Primary melanoma, VGP	BRAF V600E	Herlyn lab, Wistar Institute
WM164	Melanoma metastasis	BRAF V600E	Herlyn lab, Wistar Institute
A375P*	Primary melanoma, VGP	BRAF V600E	ATCC
SK-MEL-2*	Melanoma metastasis	NRAS Q61R	ATCC[10]
SK-MEL-19*	Melanoma metastasis	BRAF V600E	MSKCC[10]
SK-MEL-28*	Melanoma metastasis	BRAF V600E	ATCC
SK-MEL-29*	Melanoma metastasis	BRAF V600E	MSKCC[10]
SK-MEL-94*	Melanoma metastasis	BRAF V600E	MSKCC[11]
SK-MEL-100*	Melanoma metastasis	BRAF V600E	MSKCC[10]
SK-MEL-103*	Melanoma metastasis	NRAS Q61R	MSKCC[10]
SK-MEL-147*	Melanoma metastasis	NRAS Q61R	MSKCC[10]
SK-MEL-173*	Melanoma metastasis	NRAS Q61K	MSKCC[10]
SK-MEL-197*	Melanoma metastasis	BRAF V600E	MSKCC[10]
501MEL*	Melanoma metastasis	BRAF V600E	ATCC[12]
MeWo*	Melanoma metastasis	WT	Hernando lab[13]
A2058*	Melanoma metastasis	BRAF V600E	ATCC[14]
WM793b*	Primary melanoma, VGP	BRAF V600E	Wistar Institute[15]
WM853*	Primary melanoma, VGP	BRAF V600E	Herlyn lab, Wistar Institute
WM1366*	Primary melanoma, VGP	NRAS Q61L	Herlyn lab, Wistar Institute
WM1361a*	Primary melanoma, VGP	NRAS Q61R	Herlyn lab, Wistar Institute
WM1552c*	Primary melanoma, RGP	BRAF V600E	Herlyn lab, Wistar Institute
WM3268*	Primary melanoma, VGP	NRAS Q61K	Herlyn lab, Wistar Institute
SK-MEL-5*	Melanoma metastasis	BRAF V600E	ATCC[10]
SK-MEL-85*	Melanoma metastasis	BRAF G469S/NRAS Q61R	MSKCC[10]
SK-MEL-187*	Melanoma metastasis	WT	MSKCC[10]
451Lu*	Melanoma metastasis	BRAF V600E	Herlyn lab, Wistar Institute

*** Cell lines used for *LOXL3* mRNA analysis by qPCR (Figure 1e). MSKCC: Memorial Sloan Kettering Cancer Center**

Supplementary Table 2

Table 2. List of common proteins identified by mass spectrometry related to DDR and mitosis in 501MEL cells overexpressing LOXL3 or LOXL3Δ.

Protein	PSM 501MEL LOXL3	PSM 501MEL LOXL3Δ	FUNCTION
NUMA1	45	64	Mitosis
UBR5	22	52	DDR
RBM14	18	20	DDR
MSH6	16	19	DDR
MSH2	12	20	DDR
SMC3	11	19	Mitosis/DDR
AKAP8L	16	12	Mitosis
SMC1A	7	19	Mitosis/DDR
AKAP8	12	13	Mitosis
UBXN1	7	13	DDR
USP9X	8	11	Mitosis
BRCA2	6	12	Mitosis/DDR
EMD	5	13	Mitosis
ARL8B	9	8	Mitosis
RHOA	7	9	Mitosis
RCF3	6	9	DDR
SNW1	6	5	Mitosis

PSM: peptide spectrum matches

Supplementary Table 3

Table 3. Table summarizing the genomic alterations found by array-CGH analyses.

Cell line	Sample analyzed (vs NTC)	Experiment and hours post-infection	CHR in which alterations were found	Gain/loss of chromosomal fragments
A375P	shL3#1	(I) 72 h	9, 14	gain
	shL3#1	(II) 72 h	2, 4, 6, 8, 10, 11, 12, 14, 17, 21, 22	loss
	shL3#1	(II) 96 h	1, 5, 6, 8, 18, 21	loss
	shL3#2	(I) 72 h	1, 2, 7, 8, 9, 13, 16, 19, 20, 22, X, Y	gain
	shL3#2	(II) 72 h	1, 2, 3, 4, 8, 11, 14, 21, 22	loss
	shL3#2	(II) 96 h	2, 3, 6, 7, 8, 13, 14, 15, 18, 22	loss
WM3211	shL3#1	72 h	4, 6, 9, 14, 21	loss
	shL3#2	72 h	5, 6, 10, 13, 21	loss
			3, 7	gain
SK-MEL-28	shL3#1	72 h	1, 2, 6, 9, 11, 13, 14	loss
	shL3#2	72 h	5, 6, 8, 10, 13, 21	loss
			3, 7	gain
SK-MEL-147	shL3#1	72 h	1, 6, 12, 15, 18	loss
			5, 16, 19	gain
	shL3#2	72 h	9, 13, 14, 15, 18	loss

CHR: chromosome; (I) and (II): two biologically independent experiments

Supplementary Table 4

Table 4. List of primary antibodies used for western blot, immunofluorescence or immunoprecipitation analyses

Protein	Species[1]	Source	Ref. number	Dilution WB/IF (Fixation)	IP
LOX	rAb	K. Csiszar	-	1:1000/--	
LOXL2	rAb	K. Csiszar	-	1:1000/--	
LOXL3	rAb	K. Csiszar	-	1:1000/--	2 µl/ml
Snail1	mMab	Cell Signaling	3895	1:500/--	
α-tubulin	mMab	Sigma	T6199	1:10.000/--	
cleaved caspase-3	rAb	Cell Signaling	9664	1:500/--	
Flag	mMab	Sigma	F1804	1:1000/1:100 (MetOH)	
β-actin	mMab	Cell Signaling	8H10D10	1:10.000/--	
c-MYC	rAb	Epitomics	ab32072	1:1000/--	
p21	mAb	Millipore	OP64	1:500	
p27	mMab	Dako	M7203	1:1000/--	
γH2AX	mMab	Millipore	05-636-5	1:1000/1:200 (PFA)	
Chk1	mMab	Cell Signaling	2G1D5	1:1000/--	
p-Chk1 (S317)	rAb	Cell Signaling	2344	1:1000/--	
Chk2	rAb	Cell Signaling	2662	1:1000/--	
p-Chk2 (T68)	rAb	Cell Signaling	2661	1:1000/--	
BRCA1	rAb	Millipore	07-434	1:500	
BRCA2	mMab	Millipore	Ab-1, clone 2	1:1000	
BRCA2	rAb	Abcam	Ab9143	1:150 (MetOH)	1-4 µg/ml
Rad51	mMab	Santa Cruz	A gift from Fdez-Capetillo	1:1000/--	
MSH2	mMab	Leica	MSH2-L-CE	1:500/--	1-4 µg/ml
AurkA	rAb	Cell Signaling	4718	1:1000/--	
Cyclin A	rAb	Santa Cruz	sc-751	1:1000/--	
Cyclin B1	mMab	Cell Signaling	4135	1:1000/--	
Cyclin D1	rMab	Dako	M3642	1:1000/--	
CDK1	rAb	Santa Cruz	sc-54	1:1000/--	
Calnexin	rAb	Cell Signaling	2679	1:1000/--	
GM130	mMAb	BD Biosciences	610822	1:700/--	
EGFR	rAb	Cell Signaling	4267	1:500/--	
H3 trimethyl	rAb	Upstate	07-473	1:1000/--	
H3	rAb	Abcam	Ab1791	1:1000/--	
Lamin A/C	mMAb	Sigma	SAB4200236	1:1000/--	
SMC1A	rAb	Abcam	ab9262	1:2000/--	1 µg/ml
NUMA1	rAb	Novus Biologicals	NB500-174	1:1000/--	1 µg/ml
p-ATM (S1981)	mMab	Rockland	200-301-400	--/1:200 (MetOH)	
53BP1	rAb	Santa Cruz	H-300	--/1:200 (MetOH)	
HSP90	rAb	Cell Signaling	C45G5	1:1000/--	
ATR	rAb	Cell Signaling	2790	1:1000/--	
p-ATR (S428)	rAb	Cell Signaling	2853	1:500/--	
P53	rAb	Santa Cruz	sc-6243	1:500/--	

WB: Western blot; IF: Immunofluorescence; IP: immunoprecipitation; PFA: paraformaldehyde; MetOH: methanol;
[1] mMab: Mouse monoclonal antibody, rAb: rabbit polyclonal antibody

Supplementary Table 5

Table 5. List of secondary antibodies used for western blot or immunofluorescence analyses

Protein	Species[1]	Source	Ref. number	Dilution WB/IF
anti-rabbit-HRP	gAb	GE Healthcare	NXA934V	1:5000/--
anti-mouse-HRP	gAb	GE Healthcare	NXA931	1:5000/--
anti-goat-HRP	dkAb	Santa Cruz	sc-2020	1:2000/--
anti-mouse-Alexa 488	gAb	Molecular Probes	M.P. A11029	--/1:700
anti-rabbit-Alexa 488	gAb	Molecular Probes	M.P. A11034	--/1:700
DAPI	------	Molecular Probes	M.P. D1306	--/1:5000

WB: Western blot; IF: Immunofluorescence; HRP: horseradish peroxidase; [1] gAb: goat polyclonal antibody; dkAb, donkey polyclonal antibody

Supplementary Videos

Supplementary videos. Video-microscopy analysis of mitotic progression.

Supplementary video 1. A375P Control thymidine release.

Supplementary video 2. A375P shLOXL3#1 thymidine release.

Supplementary video 3. A375P shLOXL3#2 thymidine release.
